# Nanoparticle-modified fishpond sediments improve metal immobilization, redox homeostasis, and stress tolerance in *Spinacia oleracea* under multi-metal exposure

**DOI:** 10.3389/fpls.2026.1849234

**Published:** 2026-06-18

**Authors:** Yaoqiang Zhu, Waqas Ahmed, Mohsin Mahmood, Jochen Bundschuh, Muhammad Akmal, Lun Tan, Sajid Mehmood, Weidong Li

**Affiliations:** 1 School of Ecology, Hainan University, Haikou, Hainan, China; 2 College of Tropical Agriculture and Forestry, Hainan University, Haikou, Hainan, China; 3 College of Resources and Environment, Shanxi Agriculture University, Taiyuan, China; 4 Faculty of Health, Engineering and Sciences, University of Southern Queensland, Toowoomba, QLD, Australia; 5 Institute of Soil and Environmental Sciences, PMAS- Arid Agriculture University, Rawalpindi, Pakistan; 6 National Marine Data and Information Service, Tianjin, China

**Keywords:** cytoskeleton dynamics, fishpond sediment remediation, heavy metal immobilization, ROS signaling, sustainable agriculture

## Abstract

Roots represent the primary interface for sensing and responding to complex soil environments, where cytoskeletal networks play a central role in coordinating stress perception, ion transport, and detoxification processes. However, the contribution of cytoskeleton-mediated mechanisms to root adaptation under combined metal stress and sediment-based remediation strategies remains poorly understood. In this study, we investigated the cytoskeleton-associated physiological and molecular responses of *Spinacia oleracea* L. grown in chromium (Cr), copper (Cu), and zinc (Zn) co-contaminated soils amended with fishpond sediments (FPS) and nanoparticle-modified sediments (FPS+ZnONPs and FPS+SiNPs). By integrating soil chemical properties with root and whole-plant responses, we evaluated metal immobilization, stress mitigation, and putative cytoskeleton-associated uptake regulation inferred indirectly from physiological, biochemical, and redox-related indicators rather than direct cytological evidence under multifactorial environmental constraints. Both FPS and nanoparticle-treated FPS significantly reduced the bioavailable fractions of Cr, Cu, and Zn, with FPS+ZnONPs exhibiting the highest immobilization efficiency. These changes were associated with decreased metal accumulation in roots and shoots, which may reflect altered ion transport and cellular detoxification responses potentially associated with cytoskeleton-related processes. Improved rhizosphere conditions enhanced photosynthetic performance, chlorophyll content, and biomass production. Notably, FPS+ZnONPs markedly increased antioxidant enzyme activities and soluble sugar levels, while reducing proline, malondialdehyde, and hydrogen peroxide concentrations, indicating restoration of redox homeostasis and possible stabilization of stress-related cellular functions. Expression patterns of stress-responsive genes further supported the activation of coordinated detoxification networks, which may indirectly interact with cytoskeleton-associated and reactive oxygen species (ROS)-related signaling pathways, although direct cytoskeletal analyses were not performed in this study. Importantly, FPS+ZnONPs substantially reduced the estimated dietary intake of Cr, Cu, and Zn through spinach consumption, demonstrating the downstream benefits of improved root detoxification for food safety. Collectively, our findings suggest that nanoparticle-modified fishpond sediments enhance plant tolerance to multifactorial metal stress by coupling soil metal immobilization with physiological and molecular stress responses, while indirectly supporting the potential involvement of cytoskeleton-associated adaptive mechanisms in complex soil systems.

## Graphical Abstract

**Figure:**
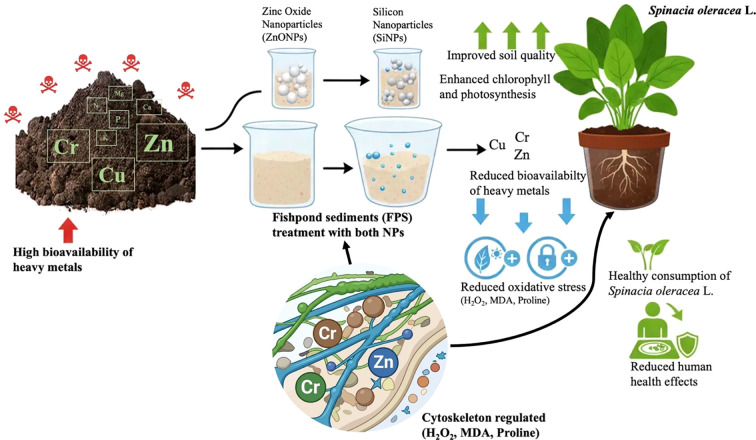
The abstract should ideally be structured according to the IMRaD format (Introduction, Methods, Results and Discussion). Provide a structured abstract if possible. If your article has been copyedited by us, please provide the updated abstract based on this version.

## Introduction

Rapid population growth and intensifying industrial activities have accelerated environmental pollution, placing substantial pressure on terrestrial and aquatic ecosystems (Chen et al., 2018; Schönbeck et al., 2022; Li et al., 2025). Among various contaminants, heavy metals (HMs) and metalloids represent a persistent global threat due to their toxicity, bioaccumulation, and long environmental residence time (Ali et al., 2019). Elevated HMs inputs from industrial effluents, urban runoff, and agricultural practices deteriorate soil and water quality, posing severe risks to plant productivity, ecosystem stability, and human health (Shaheen et al., 2020; Khan et al., 2021; Crișan et al., 2024). In soils, metal mobility and plant uptake are strongly influenced by pH, cation exchange capacity, organic matter, and drainage conditions (Kaplan et al., 2005). These interactions often lead to substantial declines in plant biomass and crop yields, amplifying economic losses and jeopardizing food security (Devkota and Schmidt, 2000; Yang et al., 2018; Sarma et al., 2021).

Given these concerns, sustainable and low-cost remediation strategies that improve soil quality and crop safety are urgently needed. Fishpond sediments (FPS), a by-product of global aquaculture expansion, have recently gained attention as a potential soil amendment. However, excessive accumulation of FPS in ponds reduces water depth, depletes oxygen levels, promotes the release of toxic gases, and suppresses aquaculture productivity (Kibria and Haque, 2018; Drózdz et al., 2020). When improperly managed, FPS can further degrade surrounding ecosystems and contribute to greenhouse gas emissions (Ma et al., 2018). Although FPS is naturally enriched with organic matter and nutrients (Han et al., 2020; Wiener et al., 2020; Kong et al., 2021), elevated HMs concentrations often limit its direct agricultural use (Haque et al., 2016; Mehmood et al., 2022a). Stabilizing these metals is therefore essential for safe FPS reutilization (Zhang et al., 2020a).

The plant cytoskeleton, composed of actin filaments and microtubules, is increasingly recognized as a central regulator of cellular responses to abiotic stress. These dynamic structures coordinate ion transport, vesicular trafficking, and intracellular signaling, thereby modulating root uptake processes and stress adaptation. Under heavy metal stress, cytoskeletal organization is often disrupted due to oxidative damage and altered calcium signaling, leading to impaired transport and loss of cellular homeostasis. Previous studies have demonstrated that heavy metals, particularly Cr(VI), can destabilize cortical microtubules and disrupt cytoskeleton-associated proteins involved in root development and cellular regulation (Adamakis and Eleftheriou, 2025). Additionally, oxidative stress generated under metal exposure may impair cytoskeleton-associated cellular organization, thereby affecting ion transport, vesicular trafficking, and root elongation processes (Adamakis et al., 2015). Conversely, stabilization of cytoskeletal dynamics has been linked to improved tolerance via enhanced detoxification, compartmentalization, and redox regulation. Despite these advances, the role of cytoskeleton-mediated mechanisms in sediment-based remediation systems, particularly under multi-metal stress conditions, remains largely unexplored (Ghuge et al., 2023; Śliwa-Cebula et al., 2023).

Nanotechnology offers an innovative, environmentally friendly approach to enhance FPS quality and remediate contaminated soils. Green-synthesized nanoparticles-produced using plant extracts (Mehmood et al., 2022b; Vijayaram et al., 2024), algae (Tadros et al., 2023), fungi (Elsayed et al., 2022), and bacteria (Mahdi et al., 2021)-are cost-effective, highly reactive, and suitable for large-scale environmental applications. ZnO nanoparticles derived from *Azolla pinnata* and Si nanoparticles extracted from *Equisetum arvense* exhibit strong adsorption and photocatalytic properties, making them efficient for HM immobilization and pollutant detoxification (El Shehawy et al., 2023; Vijayaram et al., 2024; Zhu et al., 2025).

The transformation of fishpond sediments into nanoparticle-modified amendments (NPs-FPS) offers a circular, low-input strategy with dual benefits: mitigation of heavy-metal stress in contaminated soils and improvement of soil physicochemical properties that directly influence the root environment. While previous studies have demonstrated the agronomic potential of remediated FPS for enhancing vegetable productivity, the underlying mechanisms-particularly those linking soil amendment, metal immobilization, and plant physiological responses-remain insufficiently understood (Mehmood et al., 2023), the extent to which nanoparticle-modified sediments regulate root responses under combined metal stress remains largely unexplored.

Therefore, this study aims to bridge soil remediation strategies with cytoskeleton-associated plant responses, as inferred from physiological, biochemical, and gene-expression indicators, by investigating how nanoparticle-modified sediments influence root detoxification, ion transport, and stress signaling under realistic multi-metal exposure conditions. Within this framework, the study evaluates the effects of nanoparticle-modified fishpond sediments (NPs-FPS) on root-mediated metal immobilization, uptake, and physiological stress responses in Spinacia oleracea L. exposed to combined Cu, Zn, and Cr contamination. Spinach was selected as a model plant due to its high nutritional value and sensitivity to root-zone metal toxicity (Murcia et al., 2020). We hypothesized that the incorporation of NPs-FPS into contaminated soils would (i) immobilize multiple metals and reduce their bioavailability in the rhizosphere, (ii) improve soil nutrient status and root functioning, and (iii) enhance plant performance by limiting metal uptake and activating coordinated detoxification and stress-tolerance responses. Through this multifactorial approach, the study aims to advance the mechanistic understanding of root responses in complex soil environments, while only indirectly inferring the involvement of cytoskeleton-associated processes rather than directly assessing cytoskeletal organization, microtubule dynamics, or actin filament regulation. Furthermore, the study proposes a sustainable and economically viable strategy for the management of metal-contaminated agroecosystems.

## Materials and methods

### Materials, chemicals, and reagents

All reagents and chemicals used in this work were of analytical grade and obtained from Fuchen Chemical Reagents (China). Fresh biomass of *Azolla pinnata* and horsetail plants was gathered from Haikou City and Shaoguan City, China, respectively. These plant materials served as biological precursors for the green synthesis of ZnO and Si nanoparticles, selected due to earlier reports demonstrating their potential for heavy-metal remediation (Mehmood et al., 2022b). The detailed process of plant extract preparation and green synthesis is provided in Supplementary Information Sections 1 and 2. Anhydrous zinc sulfate (ZnSO_4_) was procured from Xilong Science Co., Ltd. (China), while hydrochloric acid (HCl) and sodium hydroxide (NaOH) were obtained from Shanghai McLean Biochemical Technology Co., Ltd. (China).

### Soil analysis and experimental setup

Farmland in Haikou, China, located at 20° 03’ 22.80” North and 110° 19’ 10.20” East, was used to collect topsoil with a depth of 0–15 cm. The fishpond sediments (FPS) were collected from Hainan, China, the collection and treatment of sediments with nanoparticles (NPs) is presented in Supplementary Information section 3. For one week, the soil was allowed to air-dry in shaded area. After this, the plant debris were removed manually, and the soil was then grounded, sieved, and kept for later tests and analyses. Subsequently, FPS and NPs+FPS were thoroughly mixed with the sieved soil at application rates of 0% and 35% (w/w). The experiment was conducted using plastic pots (10 cm diameter × 13 cm height), each filled with 500 g of soil. Prior to amendment application, the soil was artificially contaminated with chromium (Cr), copper (Cu), and zinc (Zn) to simulate multi-metal stress conditions.

Spinach (*Spinacia oleracea* L.) seeds (Chun Xia Qiu Dong Zhong Ye, Hengyang, Hunan, China) were surface-sterilized and sown in the prepared pots. After germination, the respective treatments were applied to each pot. Seedlings were irrigated as needed (typically three times per week) and thinned to maintain two uniform plants per pot. Pots without FPS or NPs+FPS amendments served as controls. The experiment followed a randomized complete block (RCB) design with three biological replicates per treatment. For growth, physiological, biochemical, and molecular analyses, plants from each replicate pot were sampled and analyzed independently. The average day/night temperatures were maintained at 25/22 °C (± 3 °C), with relative humidity controlled at 65 ± 5%. After 45 days of sowing, various morphological and biochemical parameters of spinach were evaluated. This study was carried out in a greenhouse at the School of Ecology, Hainan University, Hainan, China, during the 2023–2024 period.

### Plant analysis

#### Growth analysis

Spinach plants from three different pots per treatment were carefully uprooted and rinsed with distilled water. Measurements were taken for root length, plant height, fresh and dry root weight, and shoot weight. Fresh weights were recorded immediately after harvesting, while dry weights were determined after the plants were oven-dried at 70 °C.

For the extraction of photosynthetic pigments, 0.25 g (fresh weight) of leaf was crushed and grounded under liquid nitrogen, followed by extraction with 15 mL of acetone. The resulting extract was centrifuged (6000 rpm, 20 min, 4 °C). Subsequently, the absorption of the supernatant was measured using a spectrophotometer (Dynamica Halo DB-20, UK) at wavelengths of 663 nm, 645 nm, and 470 nm for chlorophyll a (chl a), chlorophyll b (chl b), and carotenoids, respectively. The measurement of chlorophyll a, b, and carotenoids was done equations given below and expressed in mg g^-1^ of fresh weight, following the method of Lichtenthaler and Wellburn (1983):


$$Chlorophyll_{a}=\frac{(19.3\times A_{663}-0.86\times A_{645})V}{100W}$$

$$Chlorophyll_{b}=\frac{(19.3\times A_{645}-3.6\times A_{663})V}{100W}$$

$$Carotenoids=100\;(A_{470})-3.27\;(mg\;Chl.a)-104\;(mg\;Chl.\;b)/227\;$$

Where, A represents the absorption at 663, 645, and 470 nm for chlorophyll a, b, and carotenoids, respectively. V stands for the volume of the supernatant, and W represents the fresh weight (g). Additionally, the green index of leaves was determined non-destructively using a chlorophyll measurement device (SPAD-502; Konica Minolta, Osaka, Japan) to measure chlorophyll concentration.

### Characteristics of photosynthesis

Gas exchange parameters were evaluated on the upper leaves of the plants in each pot, including leaf photosynthetic activity (Pn), transpiration rate (E), and internal CO_2_ concentration (Ci). These measurements were taken using a Portable Photosynthesis System (Li-Cor 6400; Li-Cor Inc., Lincoln, NE, USA).

### Cytoskeleton-associated interpretation framework

Although direct visualization of cytoskeletal structures was not performed, cytoskeleton-associated processes were indirectly inferred from physiological, biochemical, and gene-expression indicators related to ion transport, oxidative stress regulation, and cellular stress adaptation. Reactive oxygen species (ROS) markers (H_2_O_2_ and MDA) together with antioxidant enzyme activities (SOD, POD, and CAT) were used as indirect indicators of oxidative stress conditions that may influence cytoskeleton-associated cellular stability (Jalmi et al., 2018). In addition, stress-responsive gene expression patterns were analyzed to identify signaling responses potentially associated with cellular detoxification and stress adaptation pathways.

### Determination of Antioxidant Enzyme Activity

The activity of superoxide dismutase (SOD) in both control and treated samples was assessed using the nitro blue tetrazolium (NBT) method, as described by (Giannopolitis and Ries, 1977). Peroxidase (POD) enzyme activity was evaluated following the procedure outlined by (Matthews, 1987), with enzyme units (EU) mg^-1^ protein used to quantify the rate of oxidized guaiacol production at 436 nm. Catalase (CAT) activity was determined by observing the reduced absorbance caused by H_2_O_2_ dissociation in the presence of catalase, following the principle outlined by (Aebi, 1984).

Ascorbate peroxidase (APX) enzyme activity was measured according to the method provided by Nakano and Asada (1981). Each unit of APX activity represents the oxidation of 1 nmol of ascorbate per minute.

### Determination of proline, soluble sugar and total protein content

The fresh leaves were macerated into a Tris buffer with a concentration of 100 mM and a pH of 8.0 to prepare extract. The extracts were separated using a centrifuge for 15 minutes at 20000 rpm at 5 °C. The leaf protein content was estimated using the Bradford standard assay (Bradford, 1976). Total leaf soluble sugar content was measured based on optical density at 485 nm using the technique defined by Rastall (1991).

Leaf proline concentration was assessed following the procedure of Bates et al. (1973) with minor adjustments. Fresh leaf tissue (0.5 g) was homogenized in 3 mL of 5% (v/v) sulfosalicylic acid and centrifuged at 9000 × g for 8 min. An aliquot of the supernatant (500 µL) was then diluted with 1 mL of distilled water and reacted with 2% ninhydrin. The reaction mixture was incubated at 95 °C for 35 min and subsequently cooled to room temperature. An equal volume of toluene was added to extract the chromophore, and absorbance was measured at 520 nm using a spectrophotometer. Proline concentration was quantified using a standard curve prepared with analytical-grade L-proline.

### Determination of hydrogen peroxide and lipid peroxidation (MDA) content

The hydrogen peroxide (H_2_O_2_) content was determined with modifications to the method by Velikova et al. (2000). Briefly, a fresh leaf sample (0.5 g) was homogenized in 5 mL of 0.1% trichloroacetic acid (TCA), and the resulting extract was centrifuged at 6952 × g for 20 minutes in 10% potassium phosphate buffer. The supernatant was gently mixed, and the absorbance was measured at 390 nm. H_2_O_2_ content was then calculated using a standard curve and expressed as μM g^-1^ FW.

Malondialdehyde (MDA) was measured using a modified method developed by Heath and Packer (1968). In brief, 0.5 g of a fresh leaf sample was homogenized in 10 mL 0.1% TCA. Following extraction, the extract was centrifuged for 7 minutes at 14000 × g. After mixing with 0.5% thiobarbituric acid (TBA) and 6 mL of 20% TCA, the supernatant (1.5 mL) was heated to 95 °C for 30 minutes and cooled in ice water. The optical density of the supernatant was measured using a spectrophotometer after centrifugation for 10 minutes at 10,000 x g.

### Analysis of metal-related genes expression

The expression of metal-associated genes in spinach plants grown in heavy metal-contaminated soil amended with FPS and NPs-FPS was assessed using quantitative real-time PCR (RT-qPCR). Total RNA was extracted from spinach leaf tissue using the RNeasy Plant Mini kit (Qiagen), and RNA concentration/purity was determined with a spectrophotometer. First-strand cDNA was synthesized using a reverse transcription kit (Qiagen). Total RT-qPCR reactions and procedures for PCR amplification conditions were conducted following the protocol described by Ikram et al. (2020). **Supplementary Table 1** contains a complete listing of the gene-specific primer sequences that were utilized in this investigation. The actin gene was used as a housekeeping gene, and relative expression levels were determined using the 2^-ΔΔCT^ method.

### Analysis of heavy metals in plants

To quantify the total Cr, Cu, and Zn contents in spinach roots and shoots, plant samples underwent a washing process with deionized water to eliminate any growth medium adhering to the soil. Dry plant material samples from each treatment were subjected to digestion in a triacid mixture (HNO_3_, H_2_SO_4_, and HClO_4_) at 80 °C until a clear solution was achieved. After cooling, the digested sample was filtered and adjusted to a volume of 50 ml using deionized water. The total Cr levels in the filtrate were determined using an Atomic Absorption Spectrometer (AAS; iCE 3000 series, Thermo Scientific UK; (Sharma et al., 2022)). For the determination of concentrations of the different metals in the current study, references from Sheldon and Menzies (2005) were consulted for Cu, and Prasad and Kundu (1995) for Zn.

### Health risk assessment

For the purpose of the health risk assessment, the average daily intake (ADI) approach was applied. This methodology was adopted by (Khan et al., 2020), and it represented the ADI of vegetables when they were consumed orally. After that, the health risk index (HRI) was computed by following the specification in the equation.


$$ADI_{oral}=\;\frac{C_{meatal}\times IR_{intake}\times C_{ft}}{BW}$$

$$HRI=\;\frac{ADI_{oral}}{RfD}$$

The parameters in the provided equations are defined as follows:

ADI (Average Daily Intake): The daily intake of a heavy metal by an individual; Cmetal: The concentration of heavy metal in spinach plants, expressed in mg kg^-1^; IRintake (Ingestion Ratio): The rate at which vegetables are consumed by an individual; Cft (Conversion Factor): The factor used to convert fresh vegetable weight to dry weight; BW (Body Weight): The average body weight of an individual, typically measured in kilograms; HRI (Health Risk Index): A measure used to assess the potential health risk associated with the intake of a contaminant; RfD (Reference Dose): The estimated daily exposure to a substance that is not expected to cause harmful effects over a lifetime.

### Correlation analysis

Soil physicochemical properties (N, P, K, Ca, Mg, pH, EC, Cr, Cu, Zn) and plant physiological variables (biomass, pigment indices, photosynthetic parameters, osmolytes, antioxidants, ROS markers, enzyme activities, and metal accumulation) were analyzed using Pearson’s correlation coefficients. Prior to analysis, all variables were tested for normality (Shapiro-Wilk test) and standardized (Z-score transformation). Pearson’s r values were computed using the “corrplot” package in R, with significance levels set at P < 0.05, P < 0.01, and P < 0.001.

### Data quality assurance and statistical analysis

To minimize the risk of contamination by Cr, Cu, and Zn, all glassware was rinsed at least three times with ultrapure water, oven-dried, and stored covered with aluminum foil until use. Laboratory personnel wore cotton coats and nitrile gloves, and all unused instruments were properly covered to maintain a clean working environment. Each treatment consisted of three independent biological replicates (n = 3), with each replicate represented by one pot containing four spinach plants. For biochemical and spectrophotometric analyses, technical measurements were performed in triplicate where applicable, and mean values were used for statistical analysis.

Statistical analyses were conducted using IBM SPSS Statistics 23. Data were first tested for normality and homogeneity of variance prior to analysis. One-way analysis of variance (ANOVA) followed by Tukey’s *post hoc* test was used to determine significant differences among treatments. Exact p-values were reported where possible, and differences were considered statistically significant at p < 0.05. Correlation and Mantel analyses were performed using the “corrplot” and related statistical packages in R software. The number of observations used in the correlation analyses corresponded to all biological replicates across treatments. To reduce false-positive interpretation during multiple comparisons, significance thresholds were evaluated at P < 0.05, P < 0.01, and P < 0.001. All figures were produced using OriginPro 9.1 b215 (OriginLab Corporation, Northampton, USA).

## Results and discussion

### Morphological characterization of nanoparticles

**Figure 1:**
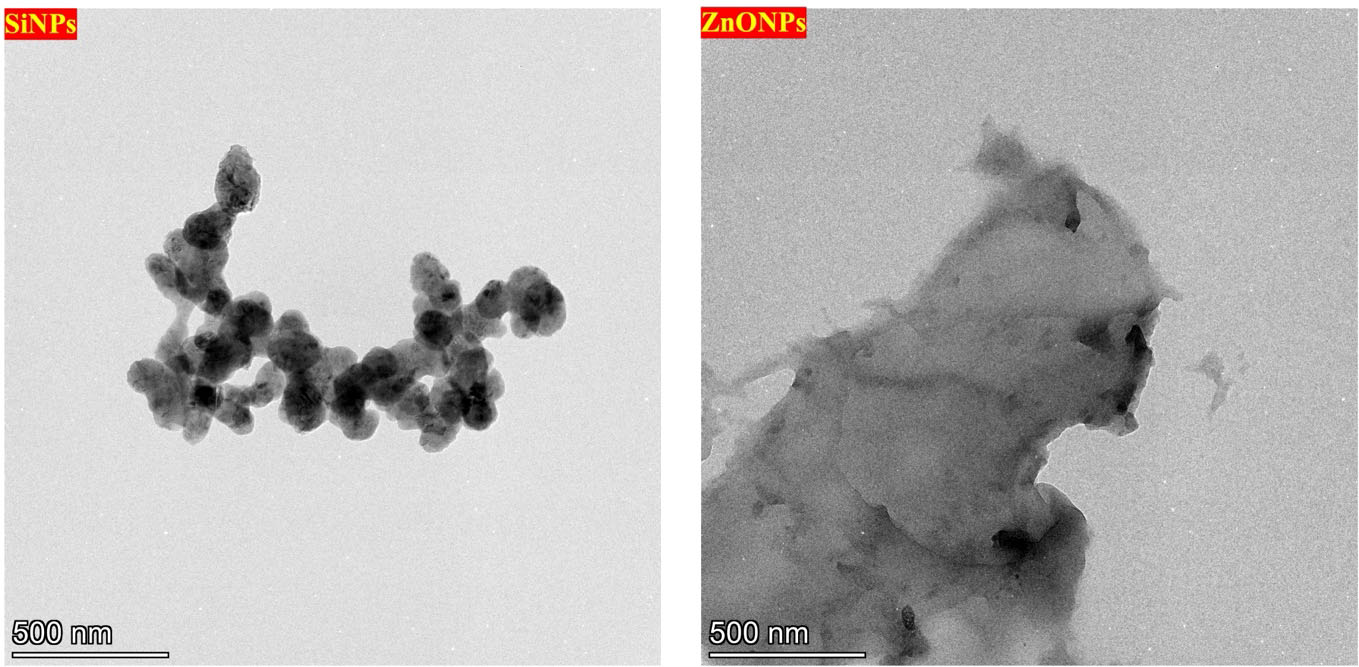
Transmission electron microscopy (TEM) images of silicon nanoparticles (SiNPs) and zinc oxide nanoparticles (ZnONPs). Scale bar = 500 nm.

The ZnO and Si nanoparticles used in this study were prepared and characterized as described in our previous publication (Zhu et al., 2025). Transmission electron microscopy (TEM) analysis revealed distinct morphological and structural differences between the synthesized silicon nanoparticles (SiNPs) and zinc oxide nanoparticles (ZnONPs) (**Figure 1**). The SiNPs exhibited a predominantly aggregated, quasi-spherical morphology, forming chain-like clusters composed of closely packed primary particles. Individual nanoparticles appear to be in the nanoscale range (tens of nanometers), although aggregation resulted in larger secondary structures (Zhang et al., 2012). This aggregation behavior is likely attributed to high surface energy and interparticle van der Waals interactions, which are commonly observed in nanoscale silicon materials (Wheeler et al., 2013). The relatively uniform contrast and compact arrangement suggest good crystallinity and particle integrity.

In contrast, ZnONPs displayed a markedly different morphology, characterized by irregular, sheet-like or flake-like structures with less defined particle boundaries. The ZnONPs appear to form amorphous or semi-crystalline aggregates, possibly resulting from particle coalescence during synthesis or drying processes (Rani et al., 2022). Compared to SiNPs, ZnONPs exhibit a more diffuse structure, indicating weaker particle-particle cohesion or a different nucleation and growth mechanism. The larger, irregular domains suggest anisotropic growth, which is typical for ZnO nanostructures synthesized under certain chemical conditions (Noimark et al., 2015). The observed morphological differences between SiNPs and ZnONPs highlight the influence of material composition and synthesis pathways on nanoparticle formation mechanisms. While SiNPs tend to form discrete spherical units followed by aggregation, ZnONPs appear to undergo particle fusion and structural spreading, leading to broader, less defined morphologies. These structural characteristics are important because they directly affect surface area, reactivity, and interaction with environmental or biological systems. These morphological variations further influence the bioavailability and stability of these materials when dispersed in diverse media, as evidenced by consistent discrepancies in hydrodynamic size and zeta potential across simulated physiological fluids (Herrera-Rodríguez et al., 2023). Furthermore, these distinct physical attributes dictate the efficacy of nanoparticle-mediated remediation, as variations in surface area directly modulate the sequestration capacity for heavy metals within complex aqueous matrices (Osman et al., 2024).

**Figure 2:**
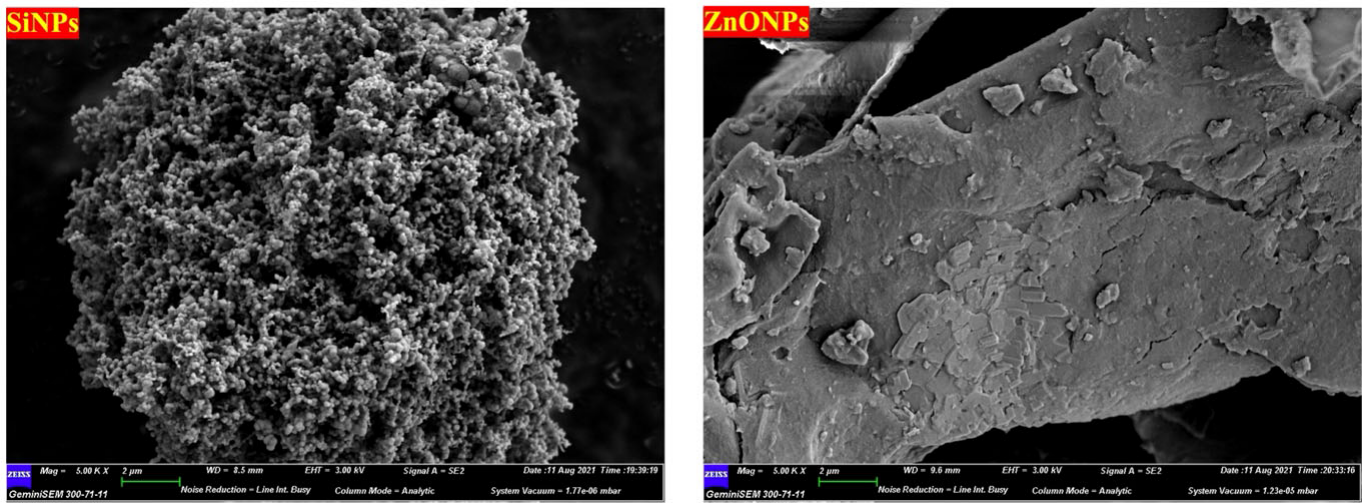
Scanning electron microscopy (SEM) images of silicon nanoparticles (SiNPs) and zinc oxide nanoparticles (ZnONPs). Scale bar = 2 µm.

Scanning electron microscopy (SEM) analysis revealed pronounced differences in surface morphology and structural organization between silicon nanoparticles (SiNPs) and zinc oxide nanoparticles (ZnONPs) (**Figure 2**). The SiNPs exhibited a highly aggregated, porous, and cauliflower-like morphology, composed of densely packed nanoscale primary particles forming spherical micro-aggregates. These aggregates appear to consist of numerous fine nanoparticles clustered together, creating a rough and highly textured surface (Johnsen et al., 2024). The particle size at the primary level is within the nanoscale range, while secondary aggregation results in larger structures in the micrometer scale (as indicated by the 2 µm scale bar). This hierarchical aggregation is typical of silicon-based nanomaterials and can be attributed to strong interparticle interactions, including van der Waals forces and possible surface oxidation effects (Wilson et al., 1993). The porous structure suggests a high specific surface area, which is advantageous for adsorption, catalytic reactions, and environmental remediation applications.

In contrast, ZnONPs displayed a markedly different morphology characterized by irregular, plate-like and fragmented structures with relatively smooth surfaces. The ZnONPs appear as larger, compact domains with evidence of particle fusion and surface flaking. Unlike the highly porous SiNPs, ZnONPs exhibit denser and less aggregated structures, indicating a different nucleation and growth mechanism during synthesis. The presence of layered or sheet-like features suggests anisotropic crystal growth, which is commonly observed in ZnO nanomaterials due to their hexagonal wurtzite crystal structure. Surface cracks and irregular edges further indicate mechanical fragmentation or post-synthesis structural rearrangement (Patil et al., 2023). The contrasting morphologies of SiNPs and ZnONPs highlight the significant role of material chemistry and synthesis conditions in determining nanoparticle architecture. SiNPs favor nucleation-dominated growth followed by aggregation, resulting in porous clusters, whereas ZnONPs exhibit growth-dominated processes, leading to larger, compact, and anisotropic structures. These differences have important implications for functional performance (Noimark et al., 2015). For instance, the high surface area of the cauliflower-like SiNPs facilitates enhanced adsorption kinetics, whereas the characteristic hexagonal wurtzite phase of the ZnONPs influences their specific photocatalytic or sensing efficacy (He et al., 2024).

### Soil immobilization mechanisms

#### Physio-chemical properties of soil

**Figure 3:**
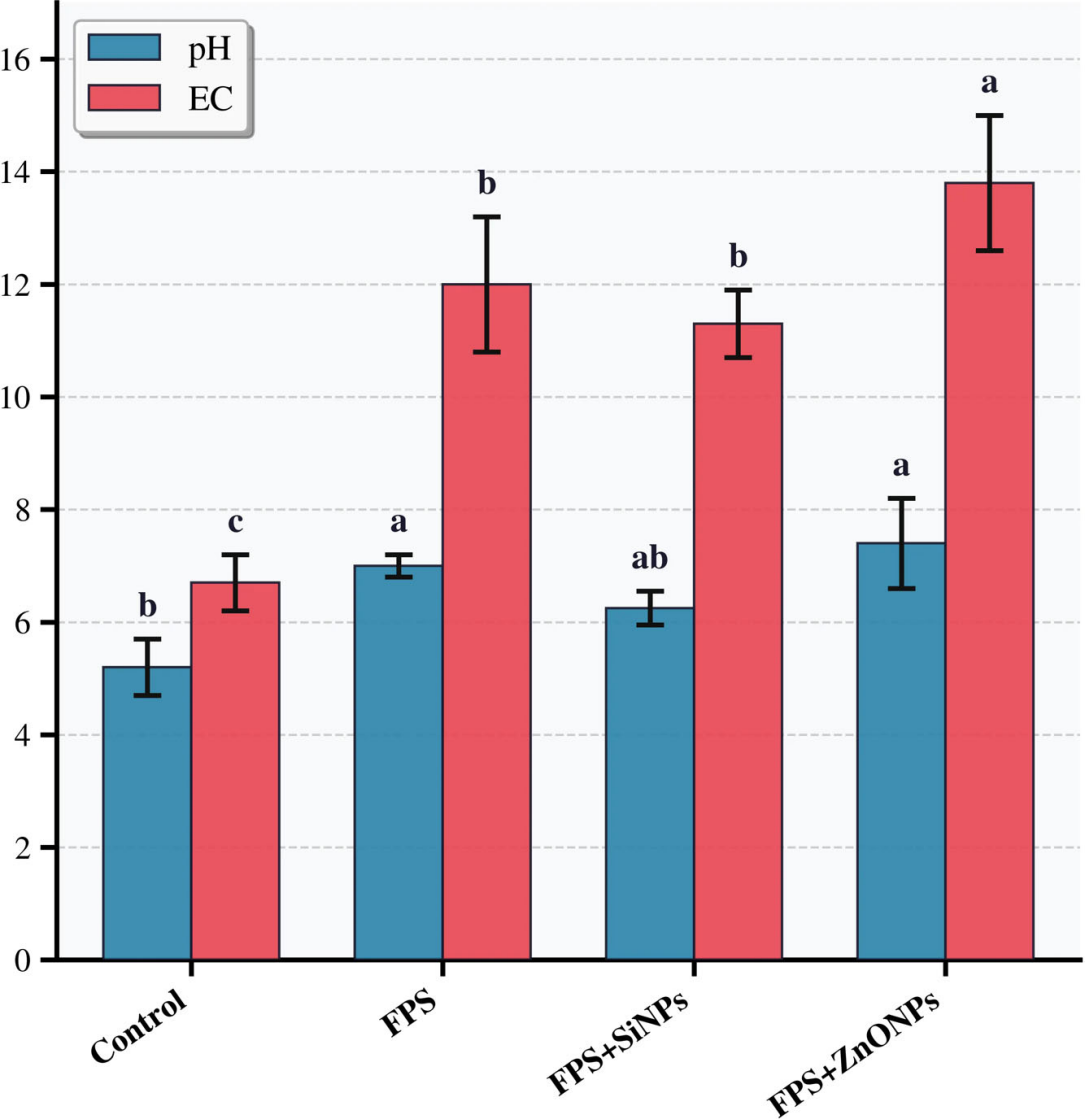
Changes in soil pH and electrical conductivity (EC) among different treatments. Control = soil without amendment; FPS = fishpond sediments applied at a rate of 35% (w/w); FPS+SiNPs = fishpond sediments amended with green-synthesized silicon nanoparticles; FPS+ZnONPs = fishpond sediments amended with green-synthesized zinc oxide nanoparticles. Values represent mean ± standard deviation (SD) of three biological replicates (n = 3). Bars with different letters indicate significant differences among treatments at p < 0.05 according to one-way ANOVA followed by Tukey’s *post hoc* test.

Roots are the first plant organs to experience abiotic constraints in contaminated soils, where combined chemical stressors directly modify rhizosphere conditions and limit crop productivity (Ullah et al., 2015). Accordingly, the use of soil passivators that stabilize the root environment has emerged as an effective strategy for mitigating metal-induced stress in agroecosystems. In this context, nanoparticle-modified fishpond sediments (NPs-FPS) represent a multifunctional amendment capable of altering soil physicochemical properties and thereby regulating root exposure to heavy metals (Mehmood et al., 2023). In the present study, application of FPS and NPs-FPS significantly altered soil pH and electrical conductivity (EC), key parameters regulating metal solubility and root uptake dynamics (**Figure 3**). Unamended contaminated soil exhibited reduced pH and EC, consistent with previous reports showing that heavy metals degrade soil chemical quality and nutrient availability (Angon et al., 2024). In contrast, amendment with FPS, FPS+SiNPs, and FPS+ZnONPs markedly increased soil pH to 6.94, 6.20, and 7.40, respectively, indicating progressive alleviation of root-zone acidity and improved ionic balance. The strongest effect observed under FPS+ZnONPs is consistent with previous studies showing that nanoparticle-enriched organic amendments enhance soil buffering capacity and promote metal immobilization in contaminated soils (Bista et al., 2019; Mehmood et al., 2022a, Mehmood et al., 2023). These shifts in rhizosphere chemistry provide a mechanistic basis for the subsequent reductions in metal bioavailability and improved root physiological performance observed under multifactorial metal stress.

Moreover, the applied amendments significantly enhanced soil nutrient status (**Supplementary Figure 1**). Nutrient enrichment was particularly evident under the FPS+ZnONPs treatment, indicating the positive contribution of NPs-FPS to soil fertility. Soil nitrogen (N), phosphorus (P), and potassium (K) increased by 24.80%, 44.37%, and 123.09%, respectively, compared to the control (**Supplementary Figure 1**). Similarly, calcium (Ca) and magnesium (Mg) levels were elevated by 44.65% and 35.67%, respectively. These improvements in nutrient availability can be attributed to the inherent nutrient content and organic matter enrichment provided by FPS, as well as the enhanced retention and stabilization effects induced by nanoparticle modification (Mehmood et al., 2022a), moreover, combining NPs with FPS further boosted the nutrient levels in FPS (**Supplementary Figure 1**). The high reactivity and small size of NPs facilitate better penetration into soil and plants, playing a crucial role in this nutrient enhancement (Tarafdar and Adhikari, 2021).

As an additional benefit, the incorporation of FPS and NPFPS led to a significant reduction in the overall concentrations of Cr, Cu, and Zn in the soil. The most significant reduction, recorded in the FPS+ZnONPs treatment (58.42%, 35.04%, and 42.73% for Cr, Cu, and Zn, respectively, relative to controls) (**Supplementary Figure 2**). The decrease in HMs levels in the soil after the NPs-FPS addition could be attributed to the binding of metal ions on the surface of amendments (Acharya and Pal, 2020), with an increased reaction rate making this method more effective than traditional *in situ* remediation methods (Alazaiza et al., 2021). Nanomaterials can improve soil structural stability by altering pore-fluid characteristics and strengthening inter-particle bonding. Their extremely small size allows them to disperse efficiently within the pore spaces of soils-particularly among fine particles that are not subjected to high compaction pressures (Singh Sekhon, 2014). Nano-based fertilizers have also been identified as a promising strategy for reducing heavy-metal (HM) contamination in soils (Prakash and Chandran, 2023). Numerous studies have demonstrated that nanoparticles can enhance the capacity of soils to immobilize or bind heavy metals, thereby limiting their mobility and bioavailability (Liu et al., 2020; Zhang et al., 2020b; Zhou et al., 2021; Keita et al., 2023; Prakash and Chandran, 2023). In the present study, application of NPs-FPS not only decreased HM concentrations but also substantially increased soil pH, electrical conductivity (EC), and both macro- and micronutrient levels. The reduction in bioavailable metal fractions suggests indirect stabilization of cytoskeleton-dependent transport processes by minimizing toxic ion interference (Ejaz et al., 2023). These findings collectively indicate that NPs-FPS amendments offer strong potential for improving soil quality and can serve as an effective option for remediation practices.

### Plant physiological responses

#### Effect on growth, photosynthesis, and metabolism

**Figure 4:**
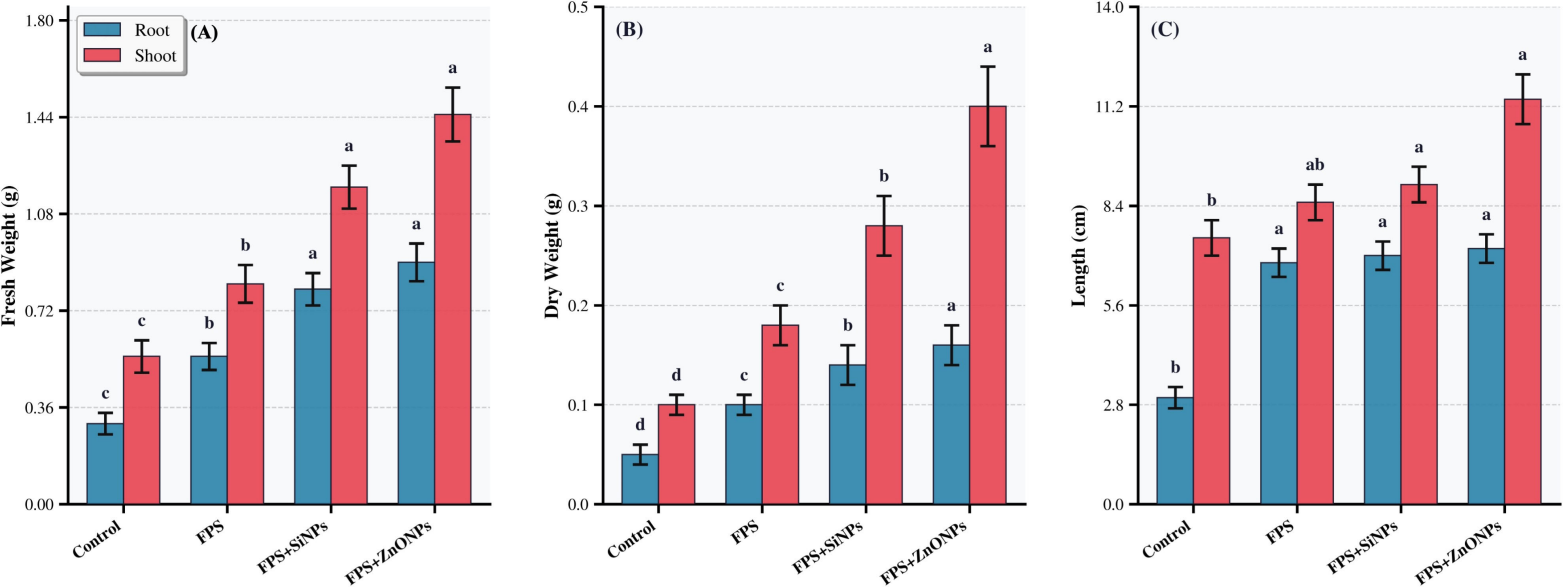
Effects of fishpond sediments treated and untreated with green-synthesized nanoparticles on the fresh weight **(a)**, dry weight **(b)**, and shoot and root length **(c)** of spinach (*Spinacia oleracea* L.) seedlings under different treatments. Control = soil without amendment; FPS = fishpond sediments applied at a rate of 35% (w/w); FPS+SiNPs = fishpond sediments amended with green-synthesized silicon nanoparticles; FPS+ZnONPs = fishpond sediments amended with green-synthesized zinc oxide nanoparticles. Values represent mean ± standard deviation (SD) of three biological replicates (n = 3). Bars with different letters indicate significant differences among treatments at p < 0.05 according to one-way ANOVA followed by Tukey’s *post hoc* test.

The addition of FPS and NPs-FPS to contaminated soil resulted in significant improvements in plant height and overall growth compared to the untreated control (**Figure 4**). In contrast, plants grown in unamended contaminated soil exhibited stunted growth, likely due to physiological and metabolic disruptions caused by heavy-metal stress. Such stress is known to impair seed germination, inhibit seedling development, and ultimately reduce plant biomass and yield (Stambulska et al., 2018). However, the application of FPS, FPS+SiNPs, and FPS+ZnONPs caused substantial increase in plant length, with improvements of 32.28%, 33.81%, and 56.44% in shoots and 108.11%, 100.40%, and 113.03% in roots, respectively. Moreover, there was a significant increase of 59.29%, 140%, and 175.50% in shoot fresh weight, while root fresh weight witnessed increments of 65.69%, 145.66%, and 185.69% in the mentioned treatments. The improvement in growth of spinach with the addition of NPs-FPS could be attributed to enhanced levels of nutrients in soil such as N, P, K, Ca, and Mg (**Supplementary Figure 1**). Moreover, it was concluded that FPS+ZnONPs was most effective amongst the treatments which improved the growth of the plants and could be recommended for similar crop production conditions.

Chlorophyll content is a crucial marker of plant productivity, and its vulnerability to metal stress can indicate damage from pollution (Areington et al., 2017). When spinach plants were exposed to Cr, Cu, and Zn stress in untreated contaminated soil, there was a reduction in chlorophyll a, chlorophyll b, and carotenoids, leading to a noticeable decrease in plant greenness, as measured by the SPAD index (**Supplementary Figure 3**). HMs stress can decrease chlorophyll content by inhibiting the enzymes involved in its biosynthesis and generating excess reactive oxygen species (ROS). These ROS cause osmotic and oxidative stress, leading to chlorophyll damage (Desoky et al., 2020). However, incorporation of FPS, FPS+SiNPs, and FPS+ZnONPs significantly increased chlorophyll a by 34.36%, 44.08%, and 67.35%, chlorophyll b by 21.29%, 45.04%, and 71.27%, carotenoids by 38.05%, 88.02%, and 272.53%, and SPAD index by 9.40%, 15.31%, and 38.86%, respectively (**Supplementary Figure 3**). The enhancement in chlorophyll content following NPs-FPS application can be attributed to the alleviation of oxidative stress (Cervantes et al., 2001). This observation is consistent with the findings of (Hussain et al., 2018), who reported that ZnO nanoparticles improved growth performance, photosynthetic activity, and grain yield in wheat exposed to heavy-metal stress. In our study, the reduction in oxidative damage was reflected by lower concentrations of malondialdehyde (MDA) and hydrogen peroxide (H_2_O_2_) in treated plants (Drózdz et al., 2020; Giannakoula et al., 2021), indicating that NPs-FPS strengthened the antioxidant defense system of spinach under heavy-metal contamination. Notably, the FPS + ZnONPs treatment produced the greatest improvements in chlorophyll a, chlorophyll b, carotenoid content, and SPAD values.

Photosynthetic parameters-net photosynthetic rate (Pn), transpiration rate (E), intercellular CO_2_ concentration (Ci), and stomatal conductance (Gs)-were evaluated in spinach grown under Cr-, Cu-, and Zn-induced stress, both with and without FPS and NPs-FPS amendments. As shown in **Table 1**, soils contaminated with Cr, Cu, and Zn caused notable declines in Pn, E, Ci, and Gs. These reductions are consistent with earlier studies; for example (Fattahi et al., 2021), documented similar decreases in photosynthetic performance under heavy-metal stress, while (Kaur et al., 2017) reported that increasing Cd levels in soil significantly reduced Pn and E in mustard plants. However, the application of FPS, FPS+SiNPs, and FPS+ZnONPs proved effective in mitigating these reductions, leading to increases in Pn by 28.21%, 35.20%, and 37.07%; E by 32.88%, 40.89%, and 52.73%; Ci by 1.19%, 2.08%, and 3.27%; and Gs by 107.08%, 182.03%, and 272.53%, respectively. Notably, the application of FPS+ZnONPs demonstrated the highest effectiveness in significantly enhancing leaf Pn, E, Ci, and Gs. This improvement in photosynthetic traits with the application FPS and NPs-FPS under higher HMs stress could be attributed to the elevated chlorophyll contents (Mehmood et al., 2021). Prop up with our findings (Abideen et al., 2020) found that amending heavy metals stressed soil positively impact photosynthetic traits.

**Table 1:** Effects of untreated and nanoparticle-treated fishpond sediments on leaf photosynthetic activity (Pn), transpiration rate (E), internal CO_2_ concentration (Ci) in spinach seedlings across different treatments.

Treatments	pn	E	Ci	Gs
Control	4.372 ± 0.656b	3.029 ± 0.337b	317.388 ± 35.348a	0.030 ± 0.003d
FPS	5.605 ± 0.369ab	4.024 ± 0.526a	313.604 ± 28.036a	0.063 ± 0.008c
FPS+SiNPs	5.910 ± 0.711a	4.267 ± 0.381a	324.001 ± 42.323a	0.085 ± 0.006b
FPS+ZnONPs	5.992 ± 0.958a	4.625 ± 0.344a	327.751 ± 22.797a	0.113 ± 0.010a

Control (without any treatments); FPS (fishpond sediments applied at the rate 35%); FPS+SiNPs (fishpond sediment amended with green synthesized silicon nanoparticles); FPS+ZnONPs (fishpond sediment amended with green synthesized zinc oxide nanoparticles). Values followed by different letters indicate significant differences at p < 0.05 according to one-way ANOVA with Tukey’s *post hoc* test.

#### Effect on antioxidant enzymes

**Figure 5:**
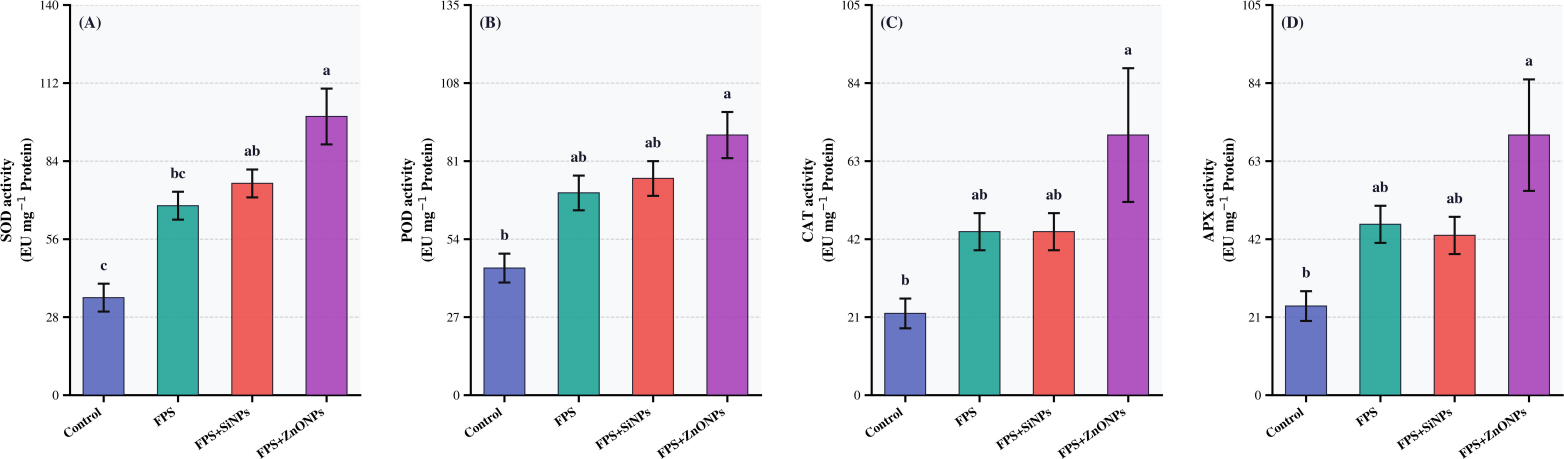
Effects of fishpond sediments treated and untreated with green-synthesized nanoparticles on antioxidant enzyme activities of spinach (*Spinacia oleracea* L.) plants, including **(a)** superoxide dismutase (SOD), **(b)** peroxidase (POD), **(c)** catalase (CAT), and **(d)** ascorbate peroxidase (APX) under different treatments. Control = soil without amendment; FPS = fishpond sediments applied at a rate of 35% (w/w); FPS+SiNPs = fishpond sediments amended with green-synthesized silicon nanoparticles; FPS+ZnONPs = fishpond sediments amended with green-synthesized zinc oxide nanoparticles. Values represent mean ± standard deviation (SD) of three biological replicates (n = 3). Bars with different letters indicate significant differences among treatments at p < 0.05 according to one-way ANOVA followed by Tukey’s *post hoc* test.

**Figure 6:**
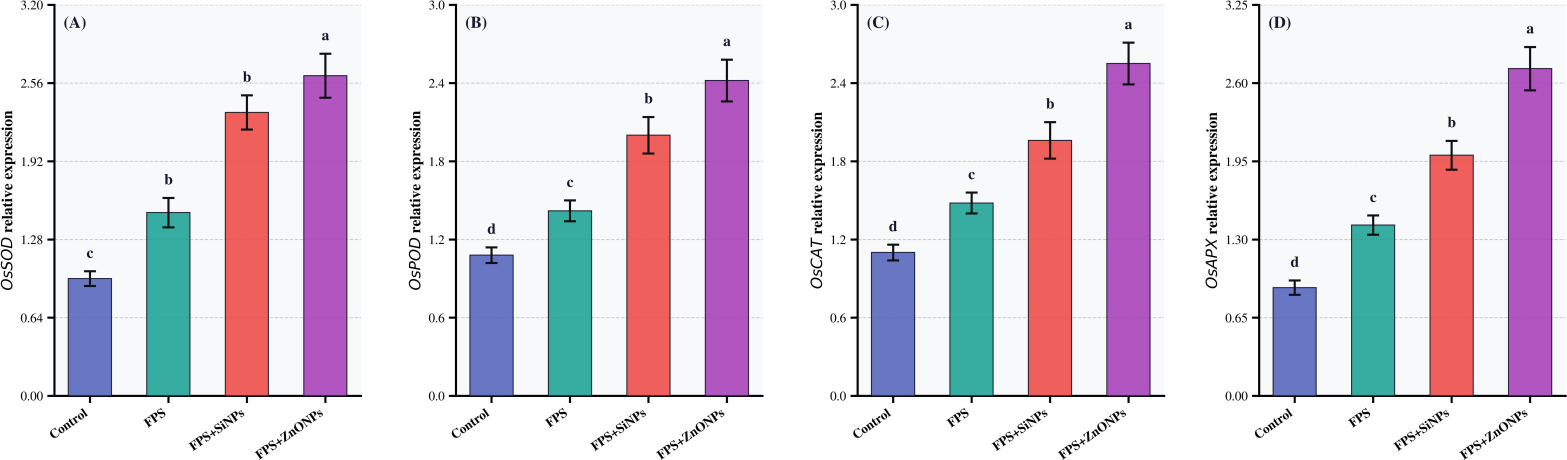
Effects of fishpond sediments treated and untreated with green-synthesized nanoparticles on the relative expression levels of antioxidant-related genes in spinach (*Spinacia oleracea* L.) plants, including **(a)** *SoSOD*, **(b)** *SoPOD*, **(c)** *SoCAT*, and **(d)** *SoAPX* under different treatments. Control = soil without amendment; FPS = fishpond sediments applied at a rate of 35% (w/w); FPS+SiNPs = fishpond sediments amended with green-synthesized silicon nanoparticles; FPS+ZnONPs = fishpond sediments amended with green-synthesized zinc oxide nanoparticles. Values represent mean ± standard deviation (SD) of three biological replicates (n = 3). Bars with different letters indicate significant differences among treatments at p < 0.05 according to one-way ANOVA followed by Tukey’s *post hoc* test.

Seedlings subjected to Cr, Cu, and Zn stress exhibited increased activity of antioxidant enzymes such as SOD, POD, CAT, and APX, which is a defense mechanism against reactive oxygen species (ROS) (**Figure 5**). Similar increases in antioxidant enzyme activities have been observed in other plants, including *Oryza sativa* and *Salvia* species (Bidabadi et al., 2020; Mehmood et al., 2021). The application of FPS, FPS+SiNPs, and FPS+ZnONPs further boosted these enzyme activities, resulting in notable improvements in SOD, POD, CAT, and APX, which contributed to better plant health. The enhancement of antioxidant enzyme activities reflects improved redox homeostasis, which is critical for maintaining cytoskeletal integrity under oxidative stress conditions (Sies and Jones, 2020). Specifically, seedlings treated with these amendments showed significant increases in SOD, POD, CAT, and APX activities, with FPS+ZnONPs showing the highest enhancements: SOD by 145.54%, POD by 120.34%, CAT by 225.62%, and APX by 196.02%, compared to untreated controls. The selected genes (*SoSOD, SoPOD, SoCAT, and SoAPX*) are key components of antioxidant defense pathways involved in ROS detoxification and redox regulation under heavy metal stress. Although these genes are not direct markers of cytoskeletal organization, previous studies suggest that oxidative stress can influence cytoskeleton-associated cellular processes, including membrane transport and vesicular trafficking (Wilson and González-Billault, 2015; Jalmi et al., 2018; Dey et al., 2022; Mansoor et al., 2023). Therefore, the observed transcriptional responses were interpreted as indirect indicators of improved cellular stress adaptation rather than direct evidence of cytoskeleton-mediated detoxification. The FPS+ZnONPs significantly upregulated the expression of antioxidant-related genes (*SoSOD, SoPOD, SoCAT*, and *SoAPX*), with fold increases of 2.6, 2.4, 2.5, and 2.7, respectively, compared to the control (**Figure 6**). Enhanced gene expression was also observed in plants treated with FPS and FPS+SiNPs, though to a lesser extent. These transcriptional responses suggest coordinated activation of detoxification pathways potentially interacting with cytoskeleton-regulated signaling networks (Yang et al., 2024). These results align with findings from Mehmood et al. (2023), indicating that FPS applications can significantly enhance antioxidant activity, gene expression, and enzyme functions, ultimately promoting improved plant growth.

#### Effect on the total protein, soluble sugar, and proline

Heavy metal contamination, specifically with Cr, Cu, and Zn, in soil negatively affected spinach seedlings by reducing total protein and soluble sugar content while increasing proline levels (**Supplementary Table 2**). Similar declines in protein and sugar levels under heavy metal stress have been documented in other plants, such as wheat and chili, as reported by (Singh and Rathore, 2019). Our research demonstrated that the treatments with FPS, FPS+SiNPs, and FPS+ZnONPs effectively increased protein and sugar levels, with the most notable improvement in the FPS+ZnONPs treatment, which resulted in a 190.53% rise in total protein and a 36.55% rise in soluble sugar compared to untreated controls. This enhancement could be due to the adaptive mechanisms of the plants to counteract stress (Younis et al., 2015). FPS+ZnONPs might reduce the toxic impact of heavy metals by immobilizing them in the rhizosphere, decreasing their availability and uptake, and restricting their movement to the plant’s aerial parts (Bashir et al., 2020). These results highlight the potential of using locally available amendments like FPS+ZnONPs for the remediation of soils contaminated with heavy metals.

Spinach plants grown in unamended Cr-, Cu-, and Zn-contaminated soils exhibited the highest proline content compared to those in amended soils (**Supplementary Table 2**), indicating that plants utilize proline as part of their adaptive response to HM toxicity. However, the application of FPS, FPS+SiNPs, and FPS+ZnONPs led to a decrease in proline levels by 40.69%, 66.60%, and 77.59%, respectively than control. Moreover, it was noted that FPS+ZnONPs was most efficient treatment for reducing the levels of proline. Proline serves several functions, including osmoregulation, acting as a free radical scavenger, protecting cytoplasmic enzymes, providing a source of nitrogen and carbon for growth post-stress, and stabilizing cellular structures (Rai et al., 2004). The application of nanoparticles (NPs) can induce structural and physical changes in plants depending on the concentration and characteristics of the NPs, with their reactivity, surface properties, and manufacturing processes playing critical roles in determining their suitability for specific plants (Khodakovskaya et al., 2012).

### Oxidative stress and gene-expression responses

#### Effect on hydrogen peroxide and malondialdehyde

**Figure 7:**
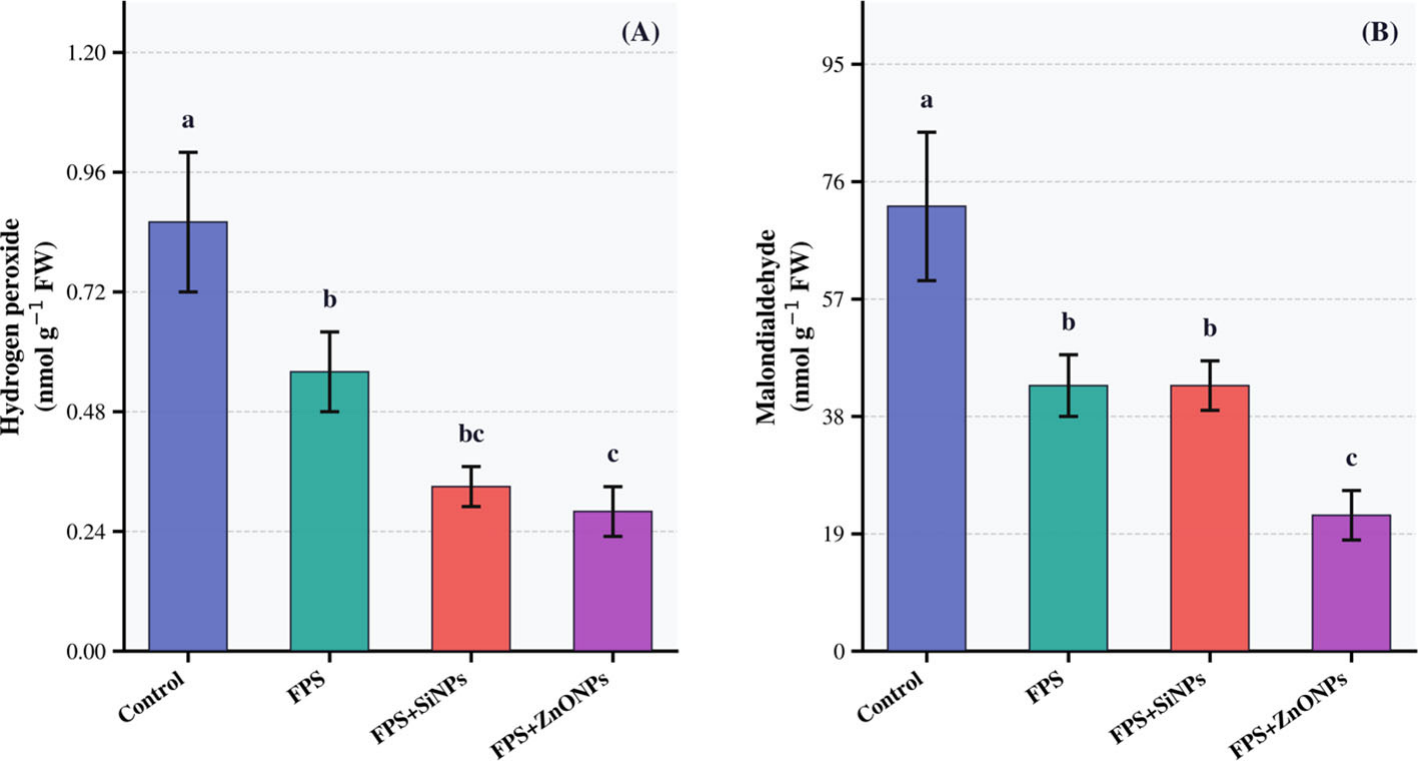
Effects of fishpond sediments treated and untreated with green-synthesized nanoparticles on **(a)** hydrogen peroxide (H_2_O_2_) and **(b)** malondialdehyde (MDA) contents in spinach (Spinacia oleracea L.) plants under different treatments. Control = soil without amendment; FPS = fishpond sediments applied at a rate of 35% (w/w); FPS+SiNPs = fishpond sediments amended with green-synthesized silicon nanoparticles; FPS+ZnONPs = fishpond sediments amended with green-synthesized zinc oxide nanoparticles. Values represent mean ± standard deviation (SD) of three biological replicates (n = 3). Bars with different letters indicate significant differences among treatments at p < 0.05 according to one-way ANOVA followed by Tukey’s *post hoc* test.

The untreated soil showed the highest accumulation of H_2_O_2_ and MDA (**Figure 7**). Elevated levels of these oxidative markers are typically associated with disruptions in the balance between reactive oxygen species (ROS) and antioxidant enzymes (Aihemaiti et al., 2020; Li et al., 2025). The extent of the increase, however, varies with metal ion concentration and plant species sensitivity (Imtiaz et al., 2018; Aihemaiti et al., 2020). In contrast, soils amended with FPS, FPS + SiNPs, and FPS + ZnONPs significantly lowered H_2_O_2_ by 36.12%, 60.79%, and 68.80%, and reduced MDA by 39.56%, 40.11%, and 69.31%, respectively, relative to the unamended control (**Figure 7**). These reductions reflect decreased lipid peroxidation and suggest that the amendments help protect cellular membranes from oxidative injury, thereby limiting MDA formation (Akhtar et al., 2012). The observed decrease in MDA and H_2_O_2_ concentrations in FPS+ZnONPs-treated spinach may be linked to the reduced levels of Cr, Cu, and Zn in the plants, as noted by (Haider et al., 2019; Sehrish et al., 2019). These findings underscore that the application of FPS+ZnONPs was the most effective treatment in reducing H_2_O_2_ and MDA levels in spinach plants subjected to Cr, Cu, and Zn stress.

### Metal uptake and root-level detoxification

#### Effect on Cr, Cu and Zn contents in the plants

As shown in **Supplementary Figure 4** the spinach plants grown in soil without amendments have higher levels of Cr, Cu, and Zn in the roots compared to the shoots. However, incorporation of FPS, FPS+SiNPs, and FPS+ZnONPs significantly reduced Cr accumulation in the shoots by 39.63%, 47.47%, and 59.32%, respectively, and in the roots by 24.92%, 38.35%, and 60.53%, respectively. Additionally, these treatments reduced Cu accumulation in shoots by 0.64, 0.57, and 0.43-folds, and in roots by 0.76, 0.63, and 0.60-folds. Moreover, they were found to be beneficial in lowering Zn accumulation both in shoots and roots of spinach plants. The decrease in the accumulation of HMs by plants in the soil after the amendment with NPs-FPS could be attributed to the binding of metal ions on the surface of the amendments, which reduces their bioavailability to plants (Mehmood et al., 2022a). The observed reduction in metal uptake and oxidative stress under nanoparticle-modified sediment treatments can be mechanistically linked to cytoskeleton-mediated regulation of root cellular processes. The cytoskeleton plays an important role in organizing membrane transporters, facilitating vesicular trafficking, and maintaining cellular polarity, all of which are associated with regulated ion uptake and intracellular compartmentalization. Under heavy metal stress, excessive ROS production can disrupt actin filaments and microtubules, leading to impaired transport functions and cellular dysfunction. Although direct cytoskeletal analyses were not performed in the present study, the observed reduction in ROS markers (H_2_O_2_ and MDA) together with enhanced antioxidant defenses may indirectly indicate improved stability of cytoskeleton-associated cellular processes through restoration of redox balance. Previous studies have reported that various nanoparticles, including selenium, silicon, titanium dioxide, and iron oxide nanoparticles, can reduce metal accumulation (e.g., cadmium and lead) in plant roots and shoots by modulating metal transporter expression and enhancing antioxidant enzyme activities such as catalase, superoxide dismutase, and glutathione peroxidase (Di et al., 2024). Enhanced antioxidant capacity can reduce oxidative damage indicators such as hydrogen peroxide and malondialdehyde, which may help minimize ROS-induced cellular disruption potentially associated with cytoskeletal organization. Additionally, nanoparticles may promote cell wall biosynthesis and iron plaque formation on root surfaces, thereby limiting metal entry under stress conditions (Di et al., 2024). Among the tested treatments, soil amended with FPS+ZnONPs showed the lowest contamination levels and reduced Cr, Cu, and Zn accumulation in both spinach shoots and roots. These findings indicate that FPS+ZnONPs could serve as a promising remediation strategy for sustainable crop production through recycling-based soil management practices.

### Integrated soil-plant interaction network

**Figure 8:**
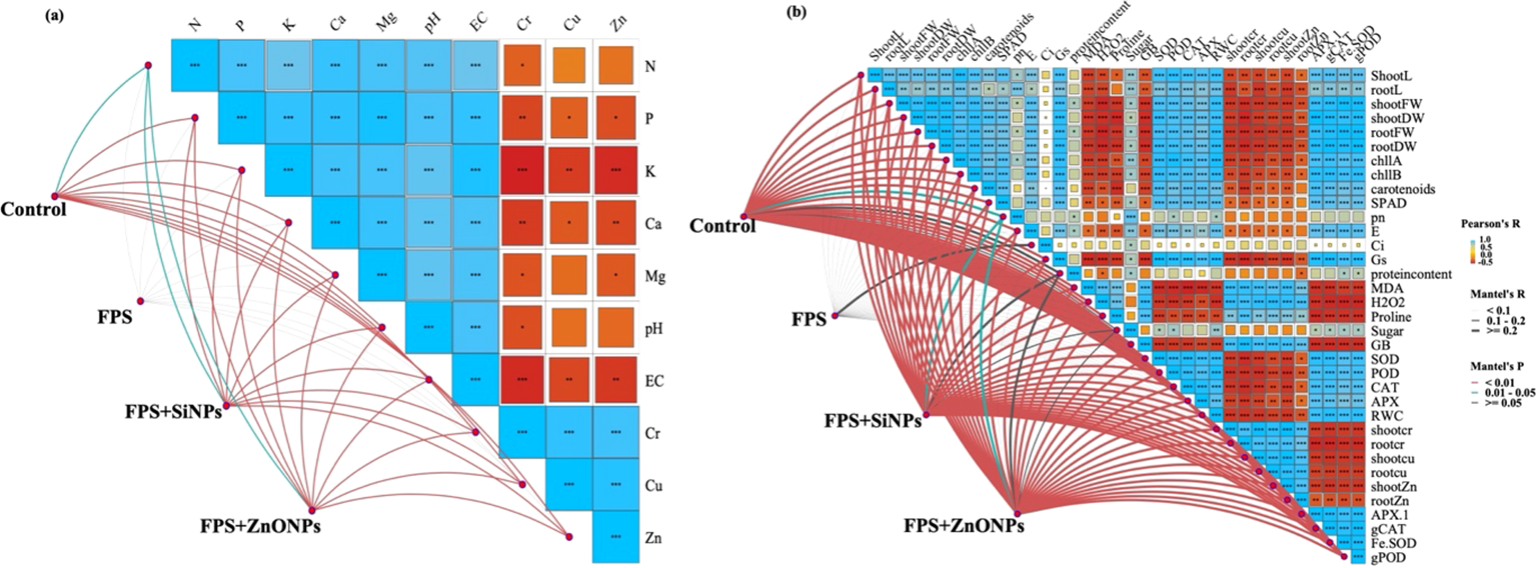
Integrated soil **(a)** and plant **(b)** correlation networks and Mantel linkages under different treatments.

The integrated correlation-Mantel analysis revealed that FPS significantly altered soil chemical behavior and plant physiological responses, forming a distinct stress-dominated interaction network, whereas the incorporation of SiNPs and ZnONPs shifted the system toward improved nutrient balance and physiological resilience. In soils (**Figure 8a**), strong positive correlations among N, P, K, Ca, and Mg, along with the tight clustering of electrical conductivity (EC) with Cr, Cu, and Zn (P < 0.001), indicate that FPS inputs created a mixed-ion environment that enhanced metal mobility. The strong EC-metal associations further suggest that increased salinity promotes ionic dissolution and co-transport of heavy metals, a pattern commonly observed in aquaculture sediments enriched with salts and organic residues (Miranda et al., 2021, Miranda et al., 2022). Mantel linkages showed FPS treatment was strongly connected to EC and heavy metals (r > 0.2, P < 0.01), whereas FPS+SiNPs weakened these associations, suggesting Si-induced precipitation or complexation reduced metal availability. FPS+ZnONPs produced the weakest linkages with Cr and Cu, indicating that ZnONPs most effectively immobilized metals, likely through surface adsorption and co-precipitation, while simultaneously lowering salinity influence (Zhu et al., 2025). The plant trait matrix (**Figure 8b**) further demonstrated that FPS exposure triggered a stress-oriented response network, where oxidative markers (MDA, H_2_O_2_) were tightly linked with antioxidant enzymes (SOD, POD, CAT, APX) and negatively associated with pigments, photosynthesis, and biomass (P < 0.001), confirming that salt-metal toxicity induced ROS formation and defense activation. In contrast, FPS+SiNPs shifted the correlation structure toward higher pigments (Chl a, Chl b, carotenoids), improved photosynthetic parameters (Pn, Ci, Gs, E), and elevated osmolytes (proline, sugars, GB), indicating improved ion homeostasis and membrane stability. FPS+ZnONPs generated the strongest positive cluster involving biomass, chlorophyll, stomatal conductance, osmolytes, and Zn accumulation, forming a dense beneficial network (r > 0.2, P < 0.01) that reflects Zn’s biochemical role in enzyme activation and oxidative stress suppression. The treatment mapping further supported these patterns, with FPS alone clustering with stress traits, Control clustering with balanced physiological attributes, and FPS+SiNPs and FPS+ZnONPs forming remediated clusters characterized by reduced metal toxicity and enhanced plant metabolism. Overall, the combined evidence demonstrates that raw FPS creates a salinity-metal toxicity interface that suppresses plant performance, while SiNPs partially and ZnONPs strongly dismantle these harmful linkages, redirecting soil-plant interactions toward nutrient efficiency, redox stability, and growth promotion.

### Human health risk implications

**Figure 9:**
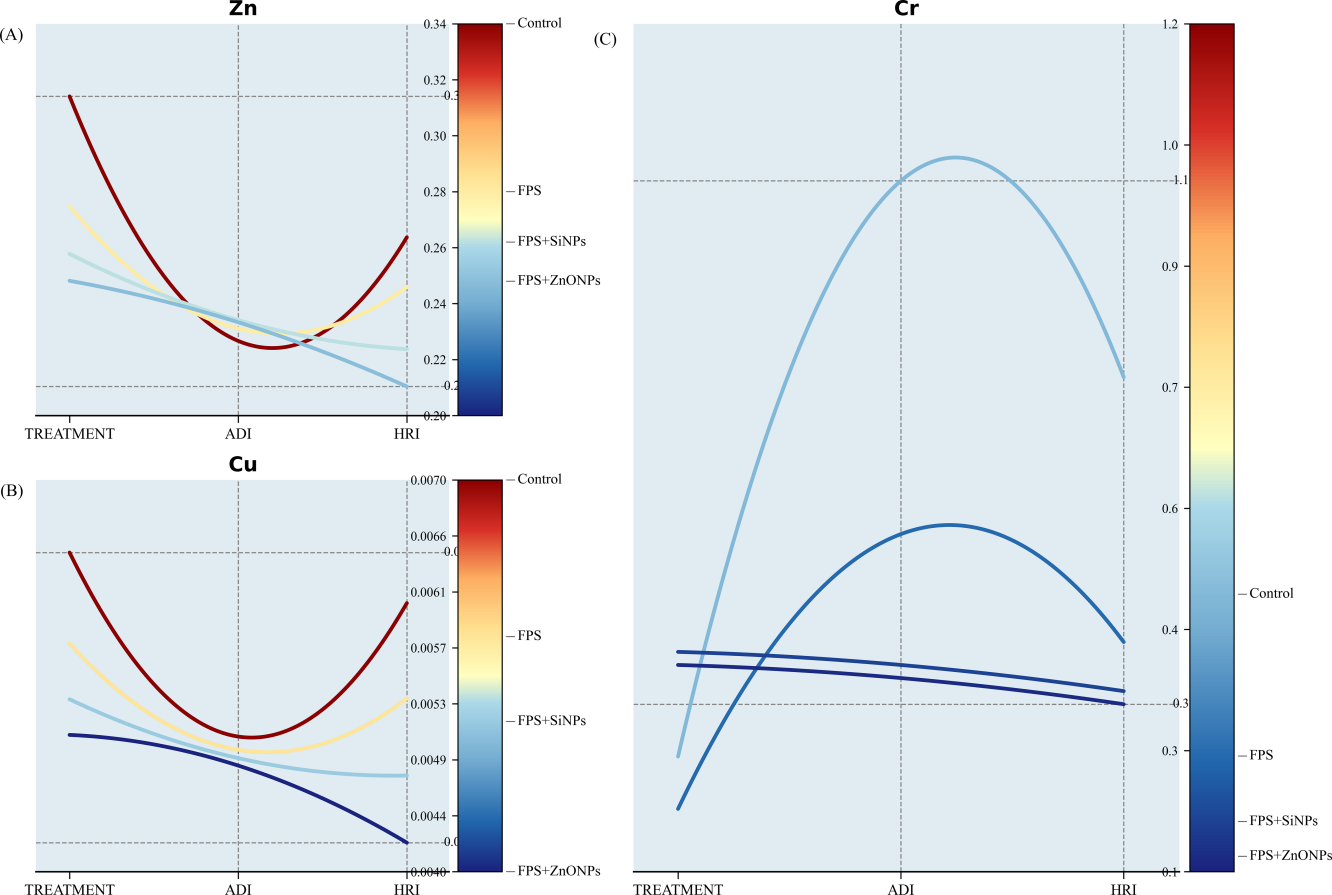
Human health risk assessment [average daily intake (ADI) and health risk index (HRI)] among different treatments.

The incorporation of NPs-FPS into contaminated soil significantly (P ≤ 0.05) reduced the average daily intake (ADI) of heavy metals in adults, with the FPS+ZnONPs treatment achieving reductions of 95.11% for Cr, 48.99% for Cu, and 44.21% for Zn (**Figure 9**). In contrast, unamended soils exhibited ADI values for Cr, Cu, and Zn exceeding the daily exposure limits established by the (EPA, 1989), indicating potential long-term health risks associated with spinach consumption. The health risk index (HRI) followed the order: Control > FPS > FPS+SiNPs > FPS+ZnONPs, demonstrating that the highest risk occurred in untreated soils, while the FPS+ZnONPs treatment effectively minimized health risks. These results are consistent with previous findings, such as those reported by Khan et al. (2020), which showed significant reductions in Cr ADI following the application of hardwood biochar.

### Proposed mechanistic framework

**Figure 10:**
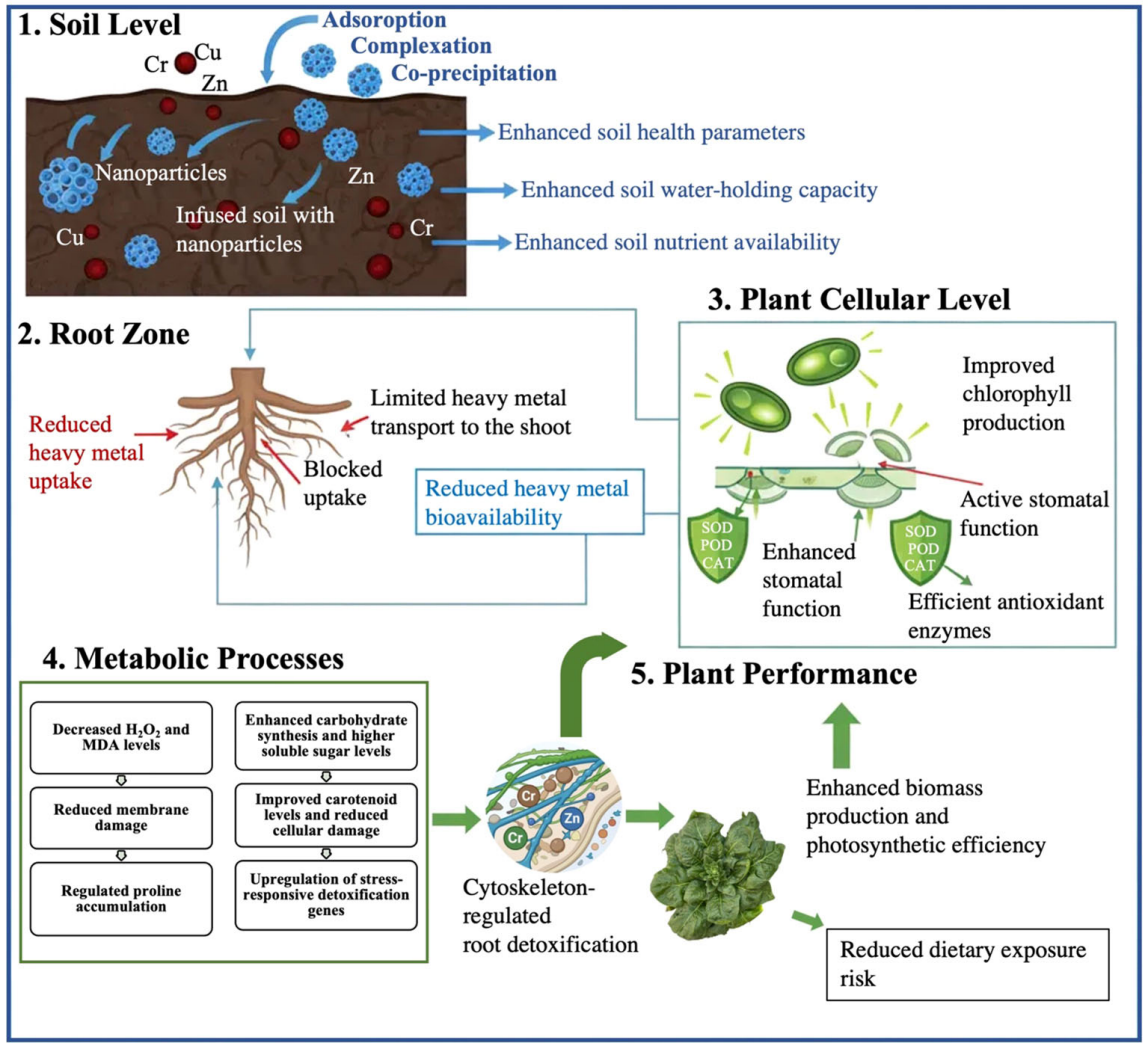
Proposed soil-plant mechanism illustrating how nanoparticle-treated fishpond sediments (NPs-FPS) reduce heavy metal toxicity and improve growth in *Spinacia oleracea*.

When fishpond sediments amended with ZnO or Si nanoparticles are incorporated into contaminated soil, they rapidly reduce the bioavailability of heavy metals (Cr, Cu, Zn) through a combination of adsorption, complexation, co-precipitation, and pH-mediated stabilization (Wang et al., 2025) (**Figure 10**). The nanoparticles possess high specific surface areas and reactive functional groups (e.g., –OH, –SH), enabling strong adsorption of metal ions through electrostatic and covalent interactions, as demonstrated by substantial reductions in DTPA-extractable Pb, Cu, Zn, Ni, and Cr following nano-silica application (Samani et al., 2024). In addition to surface sorption, both ZnO and Si nanoparticles interact with organic ligands present in sediments to form stable metal-ligand complexes, while silicon further participates in the formation of silicate structures that trap metals and decrease their solubility or mobility (Jan et al., 2024). Co-precipitation also contributes significantly to immobilization, as nanoparticles promote the formation of metal hydroxides, oxides, and silicates-such as Pb-, Cd-, and Zn-silicates-incorporating metals into mineral matrices that plants cannot readily access. These interactions are reinforced by changes in soil chemical conditions: ZnO nanoparticles, being slightly alkaline, elevate soil pH and promote the precipitation of metal hydroxides and carbonates, while both ZnO and Si nanoparticles influence soil redox status and stimulate the formation of Fe/Mn oxides that further adsorb metals (Zhang et al., 2020b). Beyond direct metal immobilization, nanoparticle-amended sediments improve soil structure, aggregation, water retention, aeration, and nutrient accessibility by enhancing cation exchange capacity and releasing essential nutrients, including Zn, N, P, and K. Furthermore, improved rhizosphere conditions may reduce the cytotoxic effects of heavy metals on cellular components, including putative cytoskeleton-associated processes, thereby supporting more efficient transport and detoxification functions. This suggests a coupled mechanism in which soil amendments regulate external metal availability while internal physiological and antioxidant responses contribute to cellular stress adaptation. Collectively, these mechanisms act synergistically to immobilize heavy metals in more stable forms, reduce their concentrations in soil solution and exchangeable pools, and limit their uptake and translocation in plants, thereby alleviating metal toxicity and promoting healthier plant growth.

## Conclusions and future implications

This study demonstrates that nanoparticle-modified fishpond sediments (NPs-FPS) enhance plant tolerance to multi-metal stress by improving soil metal immobilization and regulating physiological and molecular stress responses. Although cytoskeleton-associated mechanisms were not directly examined, the observed improvements in redox balance, reduced metal uptake, and enhanced antioxidant activity indirectly suggest the potential involvement of cytoskeleton-related adaptive processes. Among the treatments, FPS+ZnONPs was particularly effective in alleviating heavy metal stress in *Spinacia oleracea*, significantly reducing the bioavailability and plant accumulation of Cr, Cu, and Zn, while promoting plant growth, photosynthetic performance, and antioxidant defense. These improvements were further supported by enhanced soil nutrient status and reduced oxidative stress, indicating improved root–soil interactions under combined metal exposure. Importantly, the marked reduction in estimated dietary intake of heavy metals highlights the potential application of NPs-FPS for safer crop production in contaminated agroecosystems.

Future research should focus on directly elucidating the molecular and cellular mechanisms underlying root metal uptake, transport, and detoxification in nanoparticle-modified systems, particularly through cytoskeletal imaging, transporter localization, and signaling pathway analysis. Additionally, long-term and field-scale studies are required to assess the persistence, ecological safety, and practical applicability of nanoparticle-treated sediments under diverse environmental conditions. Such efforts will strengthen the mechanistic understanding and support the sustainable deployment of nanoparticle-modified fishpond sediments for the remediation of metal-contaminated soils.

## Data Availability

The original contributions presented in the study are included in the article/**Supplementary Material**, further inquiries can be directed to the corresponding author/s.
