# Supplementary material for: Nanoparticle-modified fishpond sediments improve metal immobilization, redox homeostasis, and stress tolerance in Spinacia oleracea under multi-metal exposure
Source: Front Plant Sci. 2026 Jun 18;17:1849234. doi: 10.3389/fpls.2026.1849234 (PMC13323029; doi:10.3389/fpls.2026.1849234)
Supplement: Supplementary file 1 [file Table1.docx]

**Supplementary information**

**Nanoparticle-modified sediments regulate cytoskeleton-mediated root detoxification and stress adaptation in Spinacia oleracea under multi-metal exposure**

**Yaoqiang Zhu^1,2^, Waqas Ahmed^1,2^, Mohsin Mahmood^3^, Jochen Bundschuh^4^, Muhammad Akmal^5^, Sajid Mehmood^1,2*^, Weidong Li^1,2*^**

^1^ School of Ecology, Hainan University, Haikou City, Hainan province, 570100, P.R China

^2^College of Tropical Agriculture and Forestry, Hainan University, Haikou, Hainan province, 570228, P.R China.

^3^College of Resources and Environment, Shanxi Agriculture University, Taiyuan, 030031, P.R China. 252013@sxau.edu.cn

^4^Faculty of Health, Engineering and Sciences, University of Southern Queensland, Toowoomba, 4350 QLD, Australia

^5^Institute of Soil and Environmental Sciences, PMAS- Arid Agriculture University Rawalpindi 46300, Pakistan

***Correspondence:** ***Sajid Mehmood****, E-mail: [drsajid@hainanu.edu.cn](mailto:drsajid@hainanu.edu.cn);* ***Weidong Li****, E-mail: [weidongli@hainanu.edu.cn](mailto:weidongli@hainanu.edu.cn).*

**Section 1: Preparation of Plant Extract**

Plant extracts were prepared following the general procedure of (Sharma et al., 2023), with minor modifications. Fresh *Azolla pinnata* and horsetail plants were thoroughly rinsed several times with ultrapure deionized water to remove dust and surface contaminants. The cleaned material was then air-dried at room temperature (25 °C) for six days until a constant weight was reached. Dried samples were finely ground using a Worbush grinder (Dongguan Huatai Electric Appliances Co. Ltd., China) for approximately four minutes. The resulting powder was heated in 100 mL of deionized water at 55 °C for 45 minutes. After heating, the mixture was filtered, and the obtained extract was used as the reducing and stabilizing agent for the green synthesis of zinc oxide nanoparticles (GS-ZnONPs) and silicon nanoparticles (GS-SiNPs).

**Section 2: Green Synthesis of Zinc Oxide and Silica Nanoparticles**

*2.1. Synthesis of ZnO Nanoparticles*

ZnO nanoparticles were synthesized from *Azolla pinnata* leaf extract using a modified version of the method described by (Jin et al., 2018). Equal volumes (20 mL each) of *Azolla pinnata* extract and 1 M ZnSO_4_ solution were mixed (ratio 1:1) and kept under continuous stirring at 70 °C for six hours. The pH of the mixture was adjusted to 12 using 2 M NaOH. Formation of ZnONPs was visually confirmed by a change in solution color from light yellow to white. The simplified reaction pathway is represented as follows:

$$Azzola Pinnata leave extract+ {ZnSO}_{4} Zn-complex$$

$$Zn-complex+ NaOH {Zn\left( OH \right)}_{2} ZnO \left( nanoparticles \right)+H_{2}O$$

The resulting white precipitate was collected and dried at 80 °C using an oven (Shanghai Yiheng-BPG-9240A, China) prior to characterization.

*2.2. Synthesis of Silica Nanoparticles*

Horsetail plants were used as the natural silica precursor. Silicon nanoparticles were synthesized following (Mohd et al., 2017) with slight modifications. The plant extract was refluxed with 1.0 N NaOH for one hour to solubilize the silica content. The primary reaction occurring during alkaline extraction may be expressed as:

$$Si \left( horsetail plant extract \right)+ NaOH {Na}_{2}{SiO}_{3}+byproduts$$

To separate silica nanoparticles from sodium silicate, 0.1 M HCl was slowly added until the pH reached 6.0, following the procedure of (Chapa-González et al., 2018). Acidification resulted in the formation of SiO_2_ as:

$${Na}_{2}{SiO}_{3}+ 2HCl 2NaCl+{SiO}_{2}+ H_{2}O$$

The precipitated SiO_2_ was washed repeatedly with ethanol and deionized water to remove residual NaCl. The suspension was centrifuged at 9000 × g for 10 minutes, and the recovered solid was dried at 50 °C for 24 hours. The resulting green-synthesized SiNPs (GS-SiNPs) were stored in sealed vials for subsequent characterization.

**Section 3: Sediment Collection and Treatment with Nanoparticles**

Sediment samples were collected in July 2024 from the Tan Niu (TN) and San Jiang (SJ) regions of Hainan, China. Using a core sampler, five parallel subsamples were taken at each site and composited to create representative samples. These were air-dried at approximately 25 °C, homogenized, and sieved. Prepared sediments were stored in airtight plastic bags until use.

For nanoparticle treatment, 100 g (dry weight) of each sediment type was mixed with 120 mL of ultrapure water (18.2 MΩ cm^-1^) in triplicate, and the suspension was allowed to stand for 24 hours in 250 mL screw-cap bottles. Subsequently, 1 g of GS-ZnONPs or GS-SiNPs was added to the corresponding sediment samples (TN and SJ), also in triplicate. A control (CK) contained sediment without nanoparticles. All bottles were tightly sealed and vigorously shaken to ensure uniform mixing. The mixtures were incubated for 90 days at 20 ± 1 °C in the dark to allow natural interaction between nanoparticles and sediments.


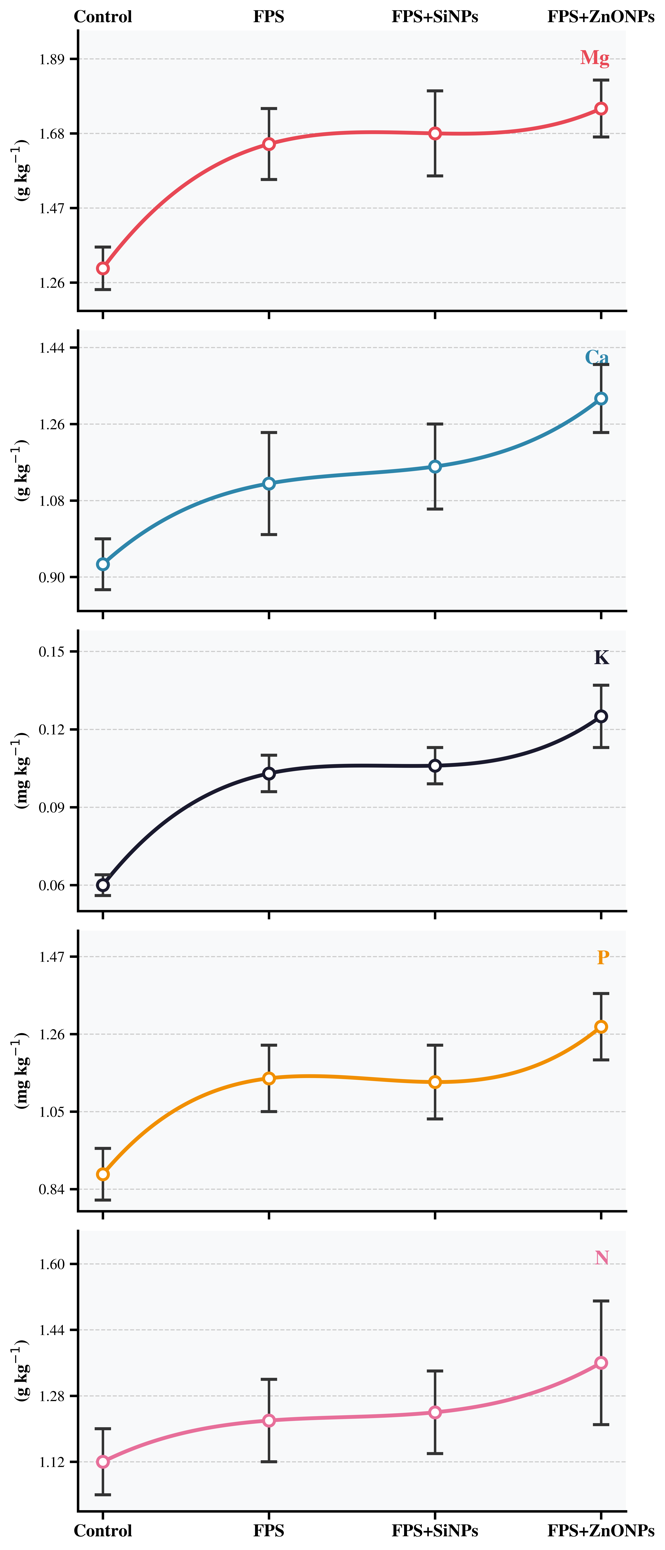


**Figure S1.** Impact of the fishpond sediments treated and untreated with green synthesized nanoparticles on the changes of total nitrogen contents, available phosphorous, exchangeable potassium, total calcium and total magnesium in soil among different treatments. Control (without any treatments); FPS (fishpond sediments applied at the rate 35%); FPS+SiNPs (fishpond sediment amended with green synthesized silicon nanoparticles); FPS+ZnONPs (fishpond sediment amended with green synthesized zinc oxide nanoparticles).


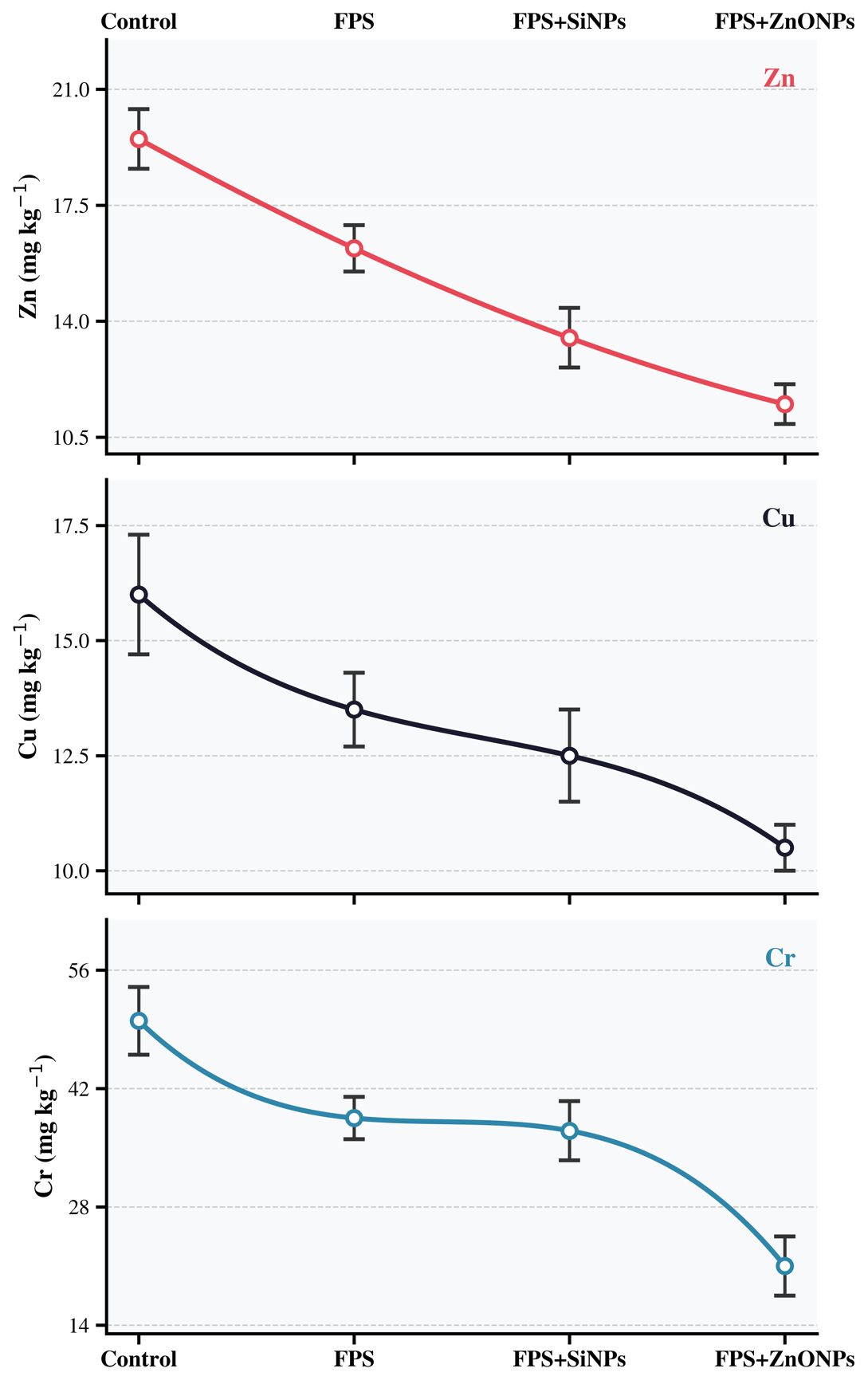


**Figure S2.** Impact of the fishpond sediments treated and untreated with green synthesized nanoparticles on the changes of total chromium, total copper and total zinc contents in soil among different treatments.


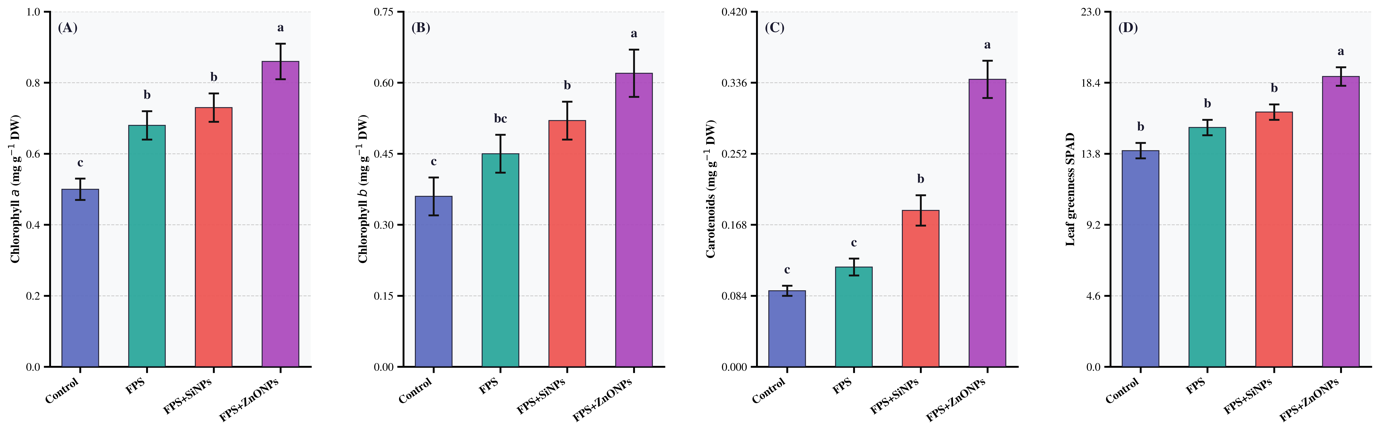


**Figure S3:** Impact of the fishpond sediments treated and untreated with green synthesized nanoparticles on the content of (a) chlorophyll a, (b) chlorophyll b, (c) carotenoids and (d) SPAD index in the spinach plants among different treatments.


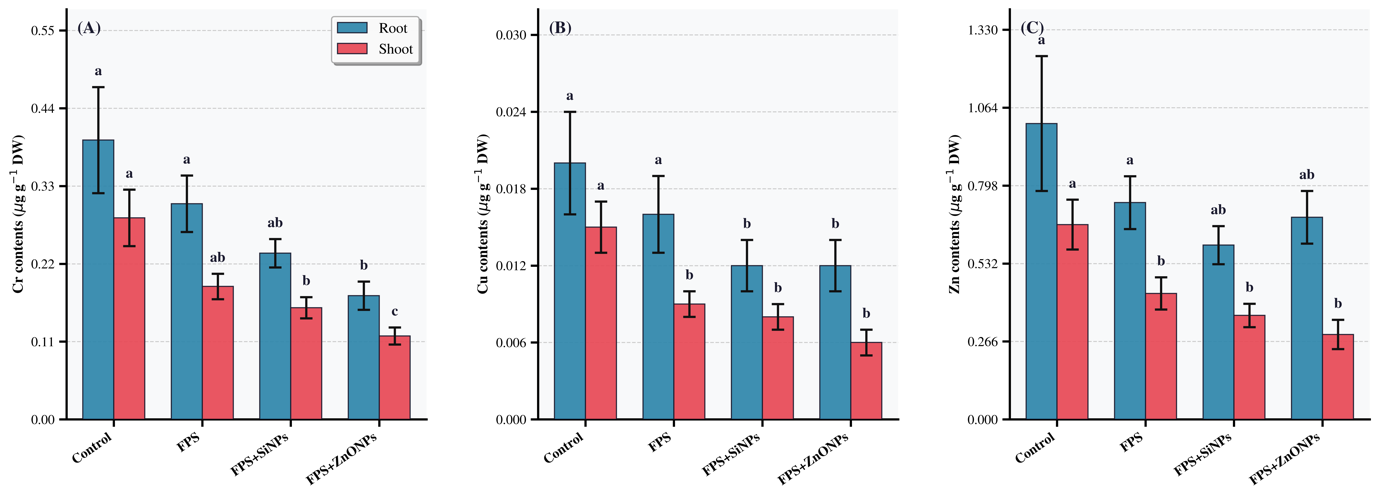


**Figure S4:** Impact of the fishpond sediments treated and untreated with green synthesized nanoparticles on Cr-, Cu- and Zn-content in the shoot and root of spinach seedlings among different treatments

**Table S1.** Gene-specific primers sequences used in the present study

| **Gene name** | **Direction** | **Gene Primer Sequence** | **References** |
| --- | --- | --- | --- |
| *SoActin* | F | GTGGTCGTACCACAGGTATC | (Ferreira et al., 2018) |
|  | R | GCCATCAAATAGAGCCAGGA |  |
| *SoSOD* | F | GAAGAGTGGCTTGTGGCATT | (Cocetta et al., 2014) |
|  | R | GCCATCAAATAGAGCCAGGA |  |
| *SoCAT* | F | CTTGATTGGCCTTTCTGTGG | (Kandlbinder et al., 2004) |
|  | R | CGAGAGACACAACAACACACA |  |
| *SoAPX* | F | CTCTCTGACCCTGTCTTCCG | (Cocetta et al., 2014) |
|  | R | CCATCAAACTCAGCAAACGA |  |
| *SoPOD* | F | ATGACTTACTACATGATGAGCTGTCC | (Li and Yi, 2012) |
|  | R | CAGTGTTGTCTTTCGTTGAATCTAG |  |

**Table S2:** Effects of untreated and nanoparticle-treated fishpond sediments on total protein, soluble sugar, and proline contents in spinach seedlings across different treatments. Values followed by different letters indicate significant differences at p < 0.05 according to one-way ANOVA with Tukey’s post hoc test.

| **Treatments** | **protein** | **Proline** | **Sugar** |
| --- | --- | --- | --- |
| Control | 0.676 ± 0.075c | 87.468 ± 10.213a | 1.998 ± 0.218b |
| FPS | 1.121 ± 0.078b | 51.881 ± 7.452b | 2.114 ± 0.147ab |
| FPS+SiNPs | 1.138 ± 0.102b | 29.210 ± 2.349c | 2.303 ± 0.082ab |
| FPS+ZnONPs | 1.965 ± 0.257a | 19.599 ± 5.355c | 2.728 ± 0.699a |

Control (without any treatments); FPS (fishpond sediments applied at the rate 35%); FPS+GSSiNPs (fishpond sediment amended with green synthesized silicon nanoparticles); FPS+GSZnONPs (fishpond sediment amended with green synthesized zinc oxide nanoparticles).

**Reference:**

Chapa-González, C., Piñón-Urbina, A. L., and García-Casillas, P. E. (2018). Synthesis of controlled-size silica nanoparticles from sodium metasilicate and the effect of the addition of PEG in the size distribution. *Materials (Basel).* 11. doi:10.3390/ma11040510.

Cocetta, G., Baldassarre, V., Spinardi, A., and Ferrante, A. (2014). Effect of cutting on ascorbic acid oxidation and recycling in fresh-cut baby spinach (Spinacia oleracea L.) leaves. *Postharvest Biol. Technol.* 88, 8–16. doi:10.1016/j.postharvbio.2013.09.001.

Ferreira, J. F. S., Sandhu, D., Liu, X., and Halvorson, J. J. (2018). Spinach (Spinacea oleracea l.) response to salinity: Nutritional value, physiological parameters, antioxidant capacity, and gene expression. *Agric.* 8. doi:10.3390/agriculture8100163.

Jin, X., Liu, Y., Tan, J., Owens, G., and Chen, Z. (2018). Removal of Cr(VI) from aqueous solutions via reduction and absorption by green synthesized iron nanoparticles. *J. Clean. Prod.* 176, 929–936. doi:10.1016/j.jclepro.2017.12.026.

Kandlbinder, A., Finkemeier, I., Wormuth, D., Hanitzsch, M., and Dietz, K. J. (2004). The antioxidant status of photosynthesizing leaves under nutrient deficiency: Redox regulation, gene expression and antioxidant activity in Arabidopsis thaliana. in *Physiologia Plantarum*, 63–73. doi:10.1111/j.0031-9317.2004.0272.x.

Li, L., and Yi, H. (2012). Effect of sulfur dioxide on ROS production, gene expression and antioxidant enzyme activity in Arabidopsis plants. *Plant Physiol. Biochem.* 58, 46–53. doi:10.1016/j.plaphy.2012.06.009.

Mohd, N. K., Wee, N. N. A. N., and Azmi, A. A. (2017). Green synthesis of silica nanoparticles using sugarcane bagasse. in *AIP Conference Proceedings* doi:10.1063/1.5002317.

Sharma, P., Kherb, J., Prakash, J., and Kaushal, R. (2023). A novel and facile green synthesis of SiO2 nanoparticles for removal of toxic water pollutants. *Appl. Nanosci.* 13, 735–747. doi:10.1007/s13204-021-01898-1.
